# The Macrophage-Osteoclast Axis in Osteoimmunity and Osteo-Related Diseases

**DOI:** 10.3389/fimmu.2021.664871

**Published:** 2021-03-31

**Authors:** Yao Yao, Xiaoyu Cai, Fujia Ren, Yiqing Ye, Fengmei Wang, Caihong Zheng, Ying Qian, Meng Zhang

**Affiliations:** ^1^ Department of Pharmacy, Women’s Hospital School of Medicine Zhejiang University, Hangzhou, China; ^2^ Department of Clinical Pharmacology, Key Laboratory of Clinical Cancer Pharmacology and Toxicology Research of Zhejiang Province, Affiliated Hangzhou First People’s Hospital, Cancer Center, Zhejiang University School of Medicine, Hangzhou, China; ^3^ Department of Pharmacy, Hangzhou Women’s Hospital, Hangzhou, China

**Keywords:** macrophages, osteoclasts, immunity, monocytes, differentiation

## Abstract

Osteoimmunity is involved in regulating the balance of bone remodeling and resorption, and is essential for maintaining normal bone morphology. The interaction between immune cells and osteoclasts in the bone marrow or joint cavity is the basis of osteoimmunity, in which the macrophage-osteoclast axis plays a vital role. Monocytes or tissue-specific macrophages (macrophages resident in tissues) are an important origin of osteoclasts in inflammatory and immune environment. Although there are many reports on macrophages and osteoclasts, there is still a lack of systematic reviews on the macrophage-osteoclast axis in osteoimmunity. Elucidating the role of the macrophage-osteoclast axis in osteoimmunity is of great significance for the research or treatment of bone damage caused by inflammation and immune diseases. In this article, we introduced in detail the concept of osteoimmunity and the mechanism and regulators of the differentiation of macrophages into osteoclasts. Furthermore, we described the role of the macrophage-osteoclast axis in typical bone damage caused by inflammation and immune diseases. These provide a clear knowledge framework for studying macrophages and osteoclasts in inflammatory and immune environments. And targeting the macrophage-osteoclast axis may be an effective strategy to treat bone damage caused by inflammation and immune diseases.

## Highlights

- Osteoimmunity is an interdisciplinary concept that refers to the interaction between the skeletal system and the immune system. Specifically, it is the interaction between immune cells and bone cells, among which the macrophage-osteoclast axis plays a fundamental role.
- In the inflammatory environment, circulating monocytes/macrophages and tissue-specific macrophages are the main sources of osteoclasts. There are three types of monocytes in the human peripheral blood: the classical (CD14^++^CD16^-^), the intermediate (CD14^++^CD16^+^), and the nonclassical (CD14^+^CD16^++^). These three phenotypes have different triggering conditions for osteoclasts differentiation.
- The fusion and multinucleation of macrophages are essential for the formation of osteoclasts to control bone mass. And the process mainly includes membrane fusion and reprogramming.
- RANKL and M-CSF are the determinants of the differentiation of macrophages into osteoclasts. PPARγ, ERRα, PGC-1β, NDUFS4, and maternal VLDLR are the regulators of the differentiation of macrophages into osteoclasts.
- In osteoporosis and other bone loss inflammatory conditions, the macrophage-osteoclast axis plays a vital role.
- The macrophage-osteoclast axis plays a crucial role in bone damage caused by inflammatory and immune diseases. And targeting the macrophage-osteoclast axis is of great significance for the treatment of bone damage.

## Introduction

The skeletal system and the immune system share multiple molecules, including cytokines, transcription factors, chemokines, receptors, and hormones. Under normal physiological conditions, the interaction between bone cells (such as osteoclasts and osteoblasts) and immune cells maintain the bone balance. Osteoimmunity is an interdisciplinary field, which is of great significance for the study of osteo-damage related diseases. Receptor activator of nuclear factor-κB (RANK), receptor activator of nuclear factor-κB ligand (RANKL), and osteoprotegerin (OPG) are the three core molecules in osteoimmunity. The ratio of RANKL to OPG determines the balance of bone resorption and bone remodeling. There are already many reviews on RANK, RANKL, and OPG, so we do not intend to introduce these three molecules here. Macrophages and osteoclasts are the focus of this article.

Macrophages are distributed in tissues throughout the body and contribute to both homeostasis and disease (1). Recently, it has become evident that some adult tissue macrophages originate during embryonic development and not from circulating monocytes (1). However, the current academic circles have different opinions on the origin of resident macrophages in tissues. For example, in RA, it is believed that the resident macrophages residing in the synovium come from monocytes in the embryo and peripheral blood. In the bone marrow, the tissue-resident macrophages are called “osteal macrophages”, which is believed to be mainly derived from mononuclear cells in peripheral blood. Osteal macrophages and osteoclasts are the results of competitive differentiation of myeloid progenitor. Under the sole stimulation of macrophages colony stimulating factor (M-CSF), myeloid progenitor differentiates into osteal macrophages. And under the dual stimulation of M-CSF and RANKL, myeloid progenitor differentiates into osteoclasts. Osteal macrophages has the potential to differentiate into osteoclasts, which is regulated by many factors.

## Osteoimmunity

**Figure 1:**
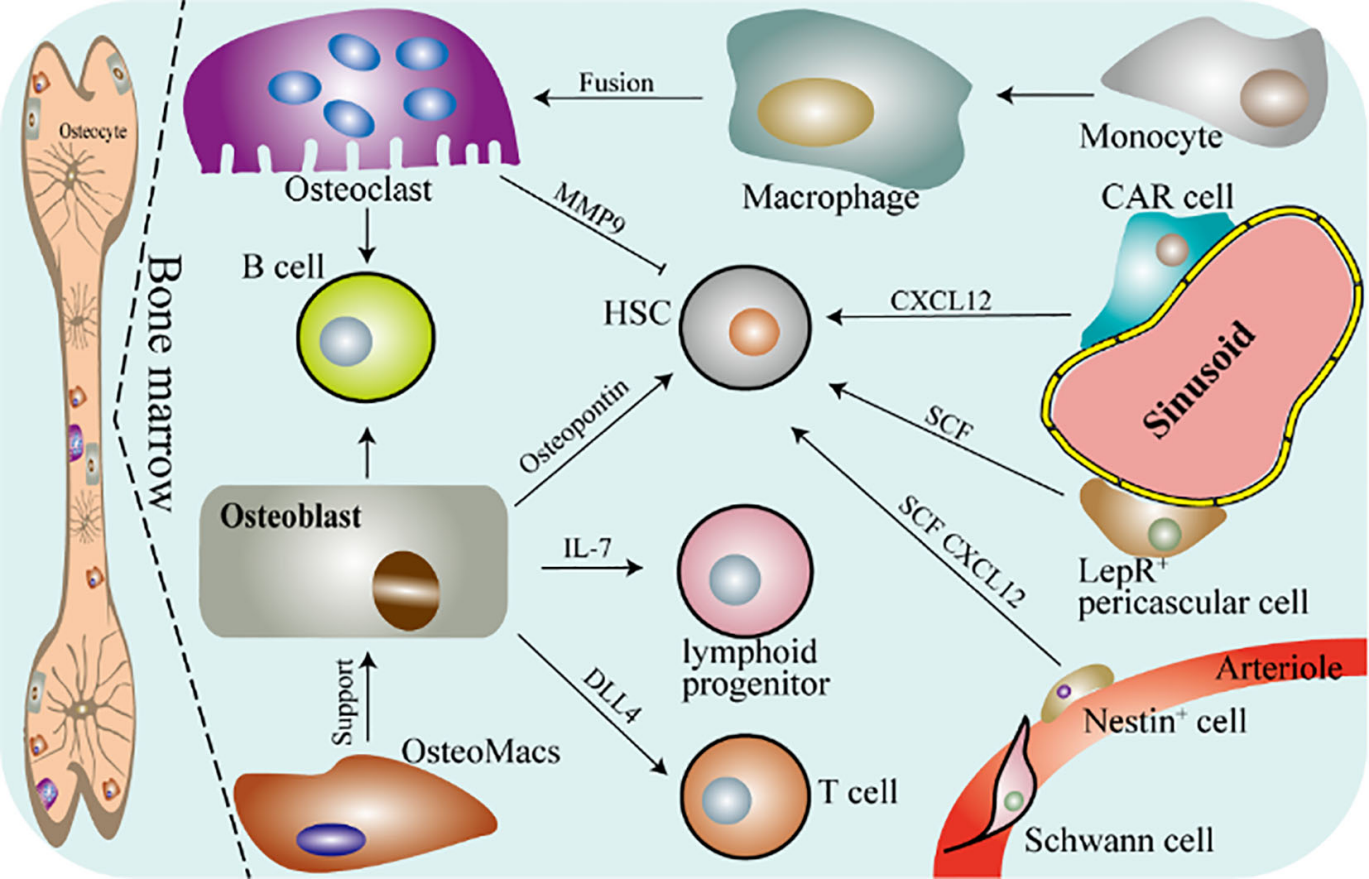
Schematic diagram of osteoimmunity. DLL4, delta-like protein 4; CAR, CXC chemokine ligand (CXCL) 12-abundant reticular; SCF, stem cell factor; HSC, hematopoietic stem cell; LepR, leptin receptor-expressing. Bone marrow mainly contains osteocytes, osteoblasts, osteoclasts, OsteoMacs and macrophages. OsteoMacs refers to the macrophages residing in the bone marrow, accounting for 15% to 20% of the total bone marrow cells. And they are mainly located near osteoblasts and support the generation of osteoblasts and bone formation. Macrophages are differentiated from monocytes derived from peripheral blood and further form osteoclasts. Bone is the cradle of immune cells, osteoclasts and osteoblasts are involved in regulating the immune response of a variety of immune cells. And they can also regulate the function of hematopoietic stem cells.

Osteoimmunity is an interdisciplinary discipline designed to elucidate the interaction between skeletal and the immune system, especially intercellular or intracellular regulation of molecular signaling in inflammatory environments such as rheumatoid joints, osteoporosis, cancer, and periodontal disease. In 2000, Aaron and Choi first proposed the concept of osteoimmunity based on the role of RANK and RANKL in inflammatory bone disease (2). The maintenance of normal bone morphology is the result of the dynamic balance of bone remodeling and bone resorption, which involves a variety of immune cells and bone cells (3). Immune cells and bone cells have a shared microenvironment and molecules, and the two cooperate to perform the function of “osteoimmunity”. The clarification of the RANKL and OPG signaling pathway has laid a solid foundation for the development of osteoimmunity, which represents that the skeletal system and the immune system are involved in both pathological and physiological conditions (2, 4, 5). Osteoimmunity is the interaction between osteoclasts or osteoblasts and various immune cells to maintain the balance of bone resorption and bone remodeling (**Figure 1**). The detection of activated T lymphocytes to express RANKL is the most direct evidence of the interaction between the skeletal system and the immune system (6, 7). Besides, overexpression of RANKL in T lymphocytes of RANKL-deficient mice can restore osteoclasts formation in the mice (8). These studies revealed that the immune system influences bone resorption by osteoclasts. Additionally, there were studies indicating that osteoclasts share regulatory molecules (including cytokines, transcription factors, chemokines, receptors, and hormones) with a variety of cells in the bone marrow (such as natural killer cell, osteal macrophages, T lymphocytes, B lymphocytes, and dendritic cells) (9–11). For example, pro-inflammatory cytokines IL-6, TNF-α, IL-1β, and IL-11 released by activated immune cells (like T lymphocytes) induce osteoclasts formation to promote bone resorption by regulating the ratio of RANKL to OPG, which is common in osteo-inflammatory diseases such as RA (12, 13). Consistent with this, the anti-inflammatory cytokines secreted by activated immune cells such as IL-4, IL-10, and interferon β (IFNβ) have the opposite effect (13, 14). The interaction between the immune system and the skeletal system is extremely complex. In general, the osteoclasts-T-lymphocytes-bone destruction pathway was once the central topic of bone immunology and has been extensively explained (9, 15). Besides, there is increasing evidence that the concept of osteoimmunity extends to osteoblasts, but it has not been well elucidated in comparison with osteoclasts (16, 17). Therefore, we do not discuss the above two branches of osteoimmunity here. In this article, we focus on the macrophage-osteoclast axis in osteoimmunity.

## The Macrophage-Osteoclast Axis

### The Macrophages Are One of the Origins of Osteoclasts

**Figure 2:**
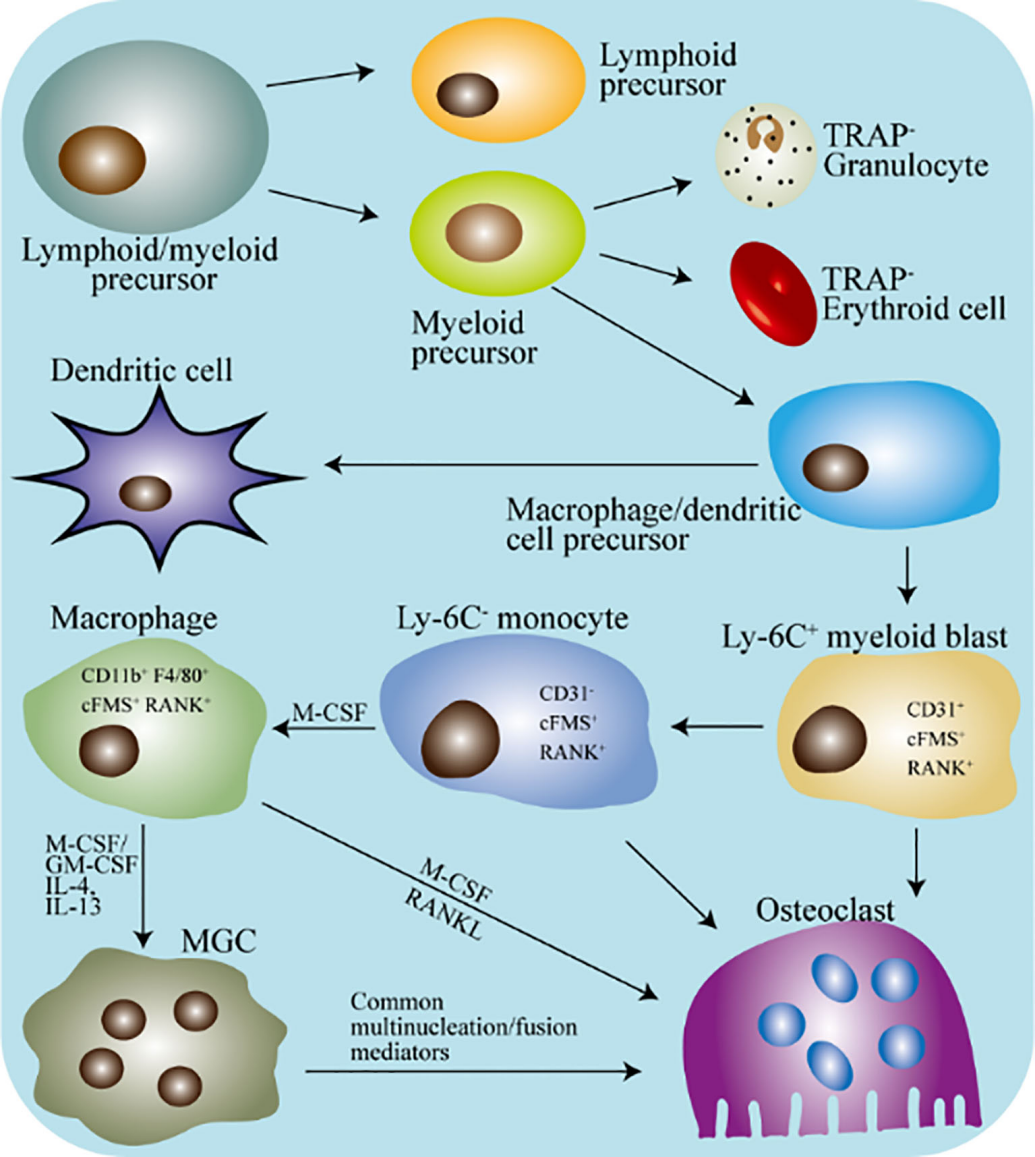
Schematic diagram of the differentiation of macrophages and osteoclasts in mice. TRAP, tartrate-resistant acid phosphatase; MGC, multinucleated giant cell; M-CSF, macrophages colony-stimulating factor; GM-CSF, granulocyte macrophages colony stimulating factor. The hematopoietic stem cells differentiate into lymphoid/myeloid precursors, and the myeloid precursors further produce dendritic cells/macrophages precursors. Under appropriate stimulation, macrophages precursors will continue to differentiate into specific marked monocytes. Next, under the stimulation of M-CSF, specifically marked monocytes differentiate into tissue-specific macrophages. Tissue-specific macrophages can directly fuse into osteoclasts, or they can fuse into MGC first and then differentiate into osteoclasts.

Osteoclasts are well-defined and distinctive in bone marrow, which originate from myeloid progenitor or osteal macrophages and is responsible for bone resorption (18, 19). An overview of the differentiation of myeloid progenitor into osteoclasts is shown in **Figure 2**. The monocytes in the peripheral blood migrate to a specific location in the bones and then fuses with each other to become mature multinucleated osteoclasts under specific stimulation (20, 21). Myeloid progenitor competitively differentiates into osteoclasts and osteal macrophages under different stimuli. Osteal macrophages can further differentiate into osteoclasts. Generally, macrophages refer to mononuclear myeloid immune cells present in most tissues, which is involved in eliminating pathogen invasion or infection, coordinating the inflammatory response, and engulfing dead cells and debris (22, 23). Typically, osteal macrophages account for about 15%~20% of the total bone marrow cells in murine (24, 25).

In 1882, Elie Metchnikoff first defined the concept of macrophages: myeloid monocytes with phagocytic properties (26). Macrophages exist in almost all tissues and is mainly involved in the immune response and inflammatory response of the tissue to maintain homeostasis. Macrophages are a part of the innate immune response and has the potential to fuse with themselves or other cells to form the multinucleated cells. As early as 1868, Langhans reported the process of multinucleation of macrophages in granulomas. Unlike most fused cells, the fusion of macrophages requires specific conditions to trigger such as severe inflammatory bone destruction disease.

Under normal circumstances, monocytes circulate in the peripheral blood, and once they enter into specific tissues, they will differentiate into macrophages. In the immune response, macrophages exhibit powerful regulatory capabilities. In different tissues, macrophages exhibit different functions, which is called the diversity or heterogeneity of macrophages (27, 28). Despite some remaining controversies, the embryonic origin of key tissue-resident macrophages populations is now fully recognized (28, 29). This represents a paradigm shift in the field and also reveals an added level of complexity to the functional heterogeneity of immune cells in the tissues. We now know that multiple populations of macrophage-like cells co-exist: in the steady state, embryonic macrophages, newborn monocyte-derived macrophages, and adult monocyte-derived macrophages function alongside one another, and in inflamed tissues adult monocyte-derived cells with features of macrophages and dendritic cells are added to the already-diverse population (28, 29). Here, we will not give a detailed introduction to the diversity and heterogeneity of macrophages, because the macrophage-osteoclast axis in bone tissue and osteoimmunity is our focus in this article.

Since its original definition in 1873, the origin of osteoclasts has not been clarified. Surprisingly, in the 1970s, Walker pioneered proof that the osteoclasts originate from hematopoietic cells (30, 31). Walker’s research showed that in a mouse model of osteopetrification lacking osteoclasts, transplantation of spleen and bone marrow cells from wild-type mice restored bone resorption in the mice. These indicated that there are progenitor cells that can differentiate into osteoclasts in hematopoietic tissue, which is essential for bone resorption (30, 31). So far, many researchers have tried to identify the precursor population of osteoclasts, but they have all failed. This may be due to the lack of surface markers to distinguish macrophages and macrophages precursors.

With the development of science and technology, in 1990, Udagawa and colleagues confirmed the monocytes/macrophages origin of osteoclasts for the first time (32). Their results showed that under the stimulation of macrophages colony stimulating factor (M-CSF), hematopoietic stem cells in the bone marrow produced monocytes/macrophages lineage (32, 33). Osteoclasts can be formed by the differentiation of immature cells of the monocytes/macrophages lineage, or by the differentiation of mature osteal macrophages (32). After that, CD31 (a glycoprotein expressed on the surface of monocytes, platelet, and neutrophil) and Ly-6C (an antigen differentially expressed on monocytes) were used as markers to separate macrophages and endothelial cells from different bone marrow cell populations (34, 35). In recent years, CD31^+^Ly-6C^+^ monocyte has been proven to differentiate into macrophages and dendritic cells (DCs), which is termed myeloid blasts (**Figure 2**) (36, 37). Besides, immature dendritic cells can differentiate into traditional DC, but they can also form osteoclasts under the stimulation of M-CSF and RANKL (38, 39).

All in all, in human peripheral blood, monocytes are divided into three subgroups according to the expression of CD14 and CD16 (surface molecules): the classical (CD14^++^CD16^-^), the intermediate (CD14^++^CD16^+^), and the nonclassical (CD14^+^CD16^++^) (40, 41). However, in the peripheral blood of mice, they are classified according to the expression of Ly6C and CD43: the classical (Ly6C^++^CD43^+^), the intermediate (Ly6C^++^CD43^++^), and the nonclassical (Ly6C^+^CD43^++^) (40, 42). Based on this theory, it has been proposed that: the classical monocytes are the main origin of osteoclasts (differentiation under normal circumstances); in inflammatory environment, the intermediate monocytes are transformed into osteoclasts with high absorption potential; the non-classical monocyte differentiate into non-resorbable osteoclasts (43–46). These indicate that the monocytes/macrophages subpopulation is the determinant of the formation and function of osteoclasts, which needs further research to clarify.

### Mechanisms of the Macrophages Fuse Into Osteoclasts

**Figure 3:**
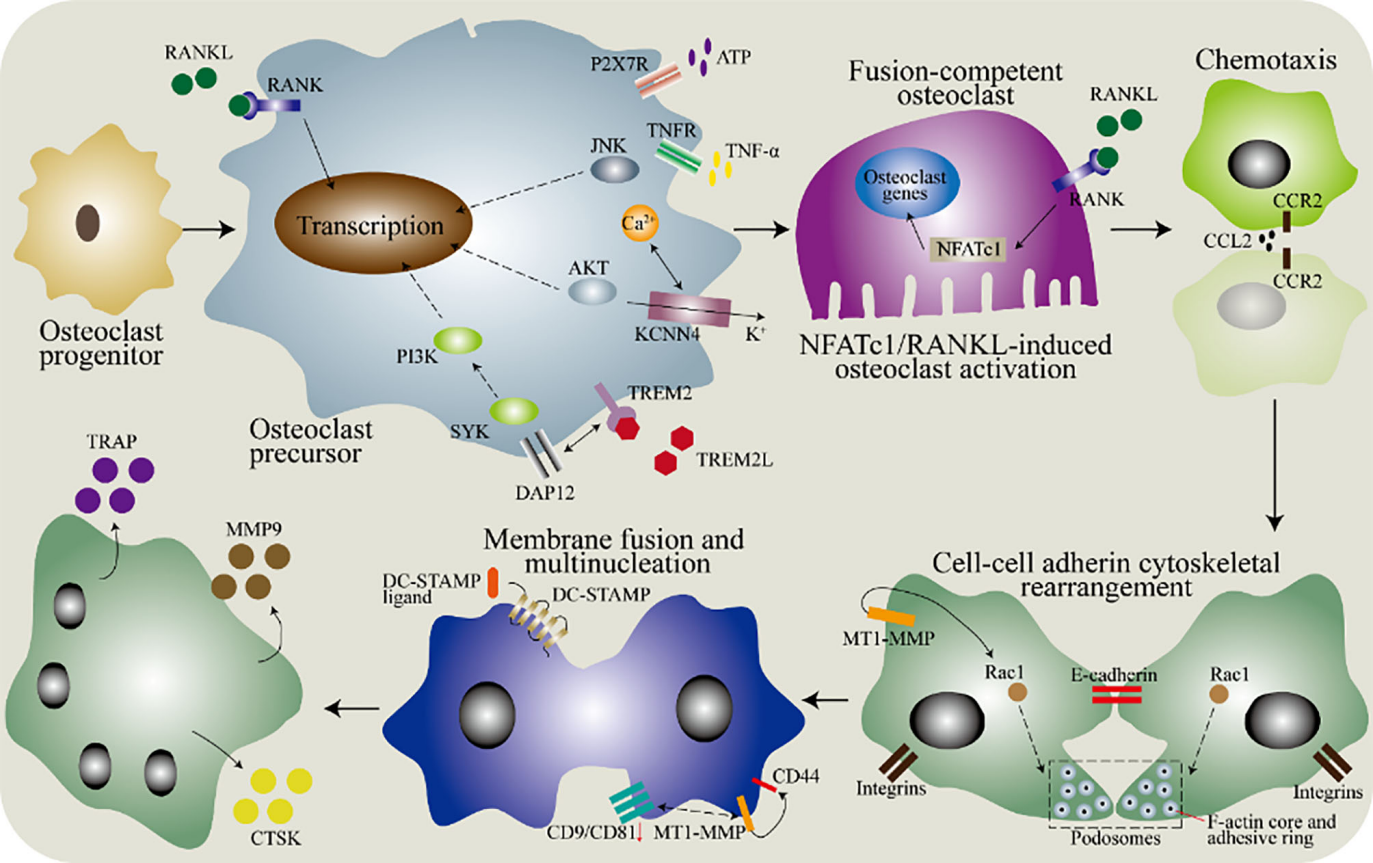
The molecular mechanism of the fusion of monocytes/macrophages into osteoclasts. In the bone marrow, osteoclasts progenitors differentiate into precursor cells after being stimulated by macrophages colony stimulating factor (M-CSF), and then various factors are needed to induce the fusion ability. In the osteoclasts precursor, DNAX activating protein 12 (DAP12) binds to triggering receptor expressed on myeloid cells 2 (TREM-2) to cause a signal *via* spleen tyrosine kinase (SYK). The purinergic receptor P2X7 (P2X7R) has a regulatory role and acts through the ATP-adenosine axis. Tumor necrosis factor (TNF) binds to the TNF receptor (TNFR) to activate the downstream c-Jun N-terminal kinase (JNK). Besides, the potassium calcium-activated channel subfamily N member 4 (KCNN4) contributes to continuous Ca^2+^ signaling and downstream activation of protein kinase B (AKT). Then, the chemotaxis of macrophages brings them close to each other, which is mediated by the binding of C-C motif chemokine ligand 2 (CCL2) to its receptor. The adhesion between cells is partially mediated by E-cadherin and integrin, and the subsequent cytoskeletal rearrangement is regulated by RAC1, which is regulated by membrane type 1 matrix metalloproteinase (MT1-MMP). Finally, the dendrocyte expressed seven transmembrane protein (DCSTAMP) mediates membrane fusion, and then forms osteoclasts through cell-cell fusion. Down-regulation of CD9 and CD81 is necessary for membrane fusion. The interaction between transmembrane protein and MT1-MMP has been determined, but further research is needed. In addition, CD44 can also mediate membrane fusion by interacting with MT1-MMP. Osteoclasts produce tartrate-resistant acid phosphatase (TRAP), matrix metallopeptidase 9 (MMP9) and cathepsin K (CTSK).

The fusion and multinucleation of macrophages are essential for the formation of osteoclasts to control bone mass (47). The fusion process includes membrane fusion and reprogramming (**Figure 3**). Many genes are involved in the process of macrophages membrane fusion, and the confirmed genes directly involved include CD47, CD44, CD81, CD9, dendritic cells stimulatory transmembrane protein (CD-STAMP), etc. (48–50). Studies indicated that DC-STAMP is directly involved in the regulation of intercellular fusion, and it is usually also used as a marker of osteoclasts fusion (48, 51, 52). However, although the role of DC-STAMP in the process of monocytes/macrophages fusion into osteoclasts has been confirmed, its ligand has still not been discovered. Interestingly, OC-STAMP (osteoclasts stimulatory transmembrane protein) mediated cell fusion does not depend on DC-STAMP, which indicates that there are other cell fusion-related proteins (53, 54). CD9 and CD81 belong to the superfamily of membrane proteins and negatively regulate the process of macrophages fusion into osteoclasts (50, 55–57). Another cell surface adhesion molecule, CD44, has been shown to be involved in regulating the adhesion and fusion of macrophages (49, 58, 59). The activity of osteoclasts is significantly inhibited through the NF-κB/NFATc1 signaling pathway after CD44 is knocked out (60). Studies by Chellaiah and Ma showed that the interaction between CD44 and membrane type 1 of matrix metalloproteinase (MT1-MMP) is one of the mechanisms of macrophages fusion (61). Furthermore, the signal transduction of Rac1 also requires the participation of MT1-MMP (62, 63).

Cell-cell fusion into multinuclei is a sign of osteoclasts differentiation and the specificity of bone tissue. The fusion of macrophages is diverse, including the fusion of macrophages and macrophages, the fusion of macrophages and monocytes, and the fusion of macrophages and other multinucleated cells (64, 65). In the bone matrix, mononuclear osteoclasts (macrophages)? also exist, but the bone resorption activity of osteoclasts is related to the number of nuclei (66, 67). The fused multinucleated cell needs to be reprogrammed to perform specific functions. The completion of the differentiation of multinucleated osteoclasts is the expression of mature markers, such as cathepsin K (CTSK), MMP9, and tartrate-resistant acid phosphatase (TRAP) (68). CTSK is a protease that can decompose gelatin, elastin, and collagen and it plays a role in decomposing cartilage and bone in bone tissue (69, 70). MMP9 is essential in the remodeling of bone and cartilage, which is necessary for the migration of osteoclasts to target tissues (71, 72). Tartrate-resistant acid phosphatase (TRAP) has now been recognized as a marker for identifying osteoclasts, because the multinucleation of osteoclasts is accompanied by an increase in TRAP staining (73). However, TRAP staining cannot distinguish mononuclear and multinucleated osteoclasts, and some activated dendritic cells or macrophages also express TRAP (74).

## Regulators of the Differentiation of Macrophages Into Osteoclasts

**Figure 4:**
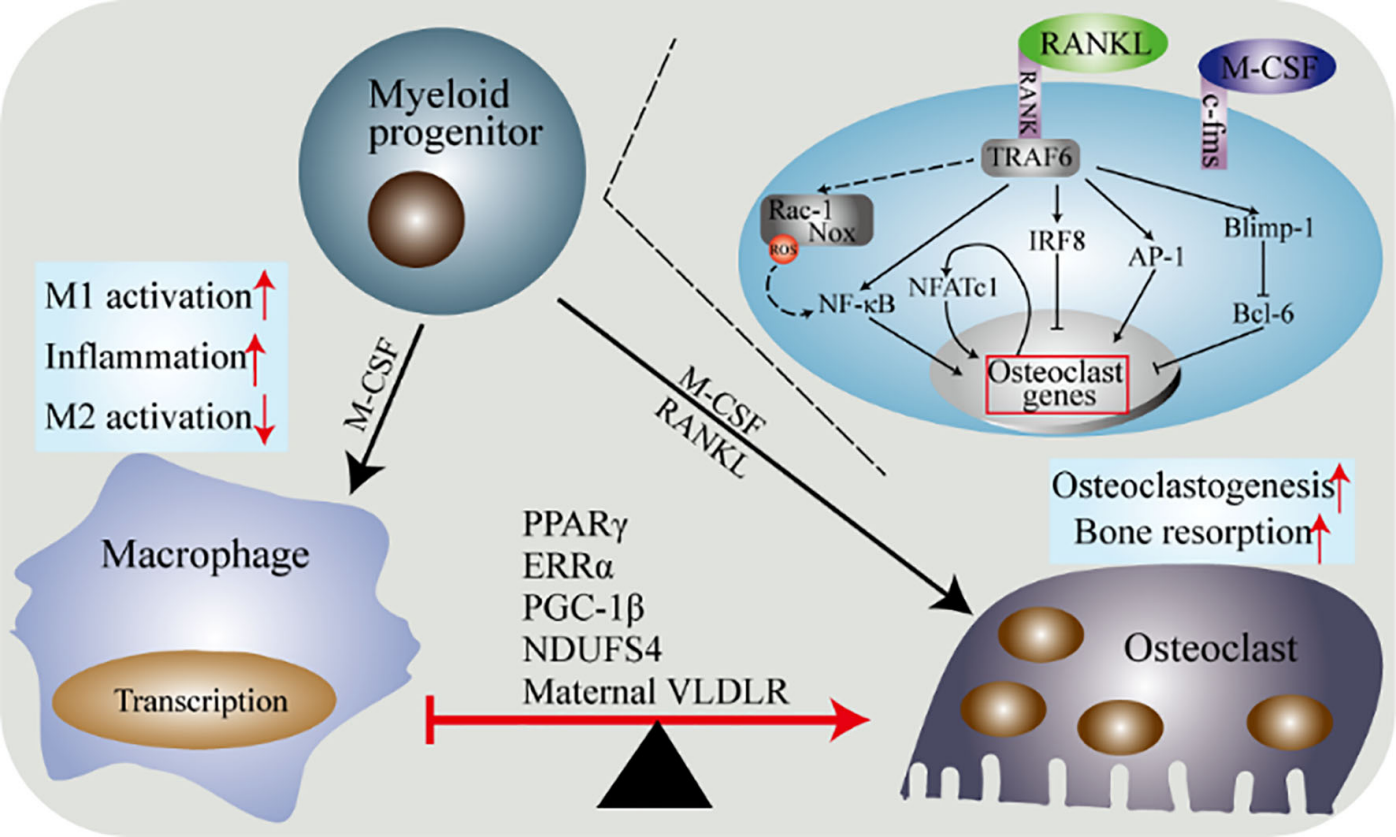
Molecular determinants of the differentiation of macrophages and osteoclasts. Macrophages and osteoclasts are the result of two competing differentiations of myeloid progenitor. Under the stimulation of M-CSF, myeloid progenitor differentiates into macrophages. Under the dual stimulation of M-CSF and RANKL, myeloid progenitor differentiates into osteoclasts. The differentiation of macrophages into osteoclasts is regulated by multiple factors. PPARγ, ERRα, PGC-1β, Ndusf4 and maternal VLDLR are currently widely recognized. The combination of RANK and its ligand and the combination of M-CSF and c-fms trigger the activation of multiple downstream pathways of osteoclasts precursor to induce osteoclasts differentiation.

Emerging evidence show that the formation of macrophages and osteoclasts is the result of competitive differentiation of myeloid progenitor. In this section, we summarize the latest research progress in the regulators that control the differentiation of macrophages and osteoclasts. These include energy metabolism, peroxisome proliferator-activated receptor γ (PPARγ) and estrogen-related receptor α (ERRα), PPARγ coactivator 1β (PGC-1β), recombinant human histidine NADH dehydrogenase Fe-S protein 4 (NDUFS4), (maternal VLDLR) (**Figure 4**).

### PPARγ

PPARγ belongs to the nuclear hormone receptor superfamily and is a ligand-inducible nuclear receptor (75), which is highly expressed in myeloidlineage cells (such as macrophages). The differentiation process of macrophages is accompanied by the expression of PPARγ (76–78), and its expression is obviously up-regulated in activated macrophages (79). However, in the differentiation process of macrophages, the specific role of PPARγ is controversial. Some studies showed that PPARγ is essential for macrophages differentiation (80, 81). However, studies based on embryonic stem cells suggested that PPARγ is not necessary for macrophages differentiation (76, 81). Later studies found that in the fetus, PPARγ only determines monocytes differentiation in the peripheral blood into alveolar macrophages, but does not affect the differentiation of macrophages in other tissues (82). Now, it is generally believed that PPARγ is a negative regulator of pro-inflammatory response and macrophage activation (79, 80). Consistent with this, in monocytes/macrophages, PPARγ agonists inhibit inflammatory signal transduction or the secretion of pro-inflammatory cytokines (such as IL-1β, IL-6, TNF-α) (79, 80, 83, 84). Besides, both animal experiments and cell experiments confirmed that the activation of PPARγ is necessary for the polarization of anti-inflammatory M2 macrophages (85, 86).

Deletion of PPARγ in osteoclasts lineage can disrupt RANK-RANKL signal transduction to inhibit the differentiation of osteoclasts (87, 88). Also, the promotion of osteoclasts differentiation by rosiglitazone depends on the activation of PPARγ (87, 89). The mechanism may be that c-fos (an important transcription factor for osteoclasts formation) is directly regulated by PPARγ (87, 90). These results confirm an important conclusion that activation of PPARγ can promote bone resorption and osteoclasts differentiation (91–93). Interestingly, monocytes chemoattractant protein-1 (MCP-1) and TNFα genes (macrophages maturation-related genes) were up-regulated in mutant osteoclasts whereas inhibited by rosiglitazone in wild-type (87, 94), which is consistent with the anti-inflammatory effect of PPARγ discussed above (79, 83). Furthermore, osteoclasts progenitor coexists with PPARγ in the bone marrow (95). In addition to osteoclasts, PPARγ was also found to inhibit osteoblasts differentiation and bone formation (96–98).

To sum up, PPAR is a critical molecular modulator in macrophages and osteoclasts, specifically promoting osteoclasts differentiation/activation but inhibiting the activation of pro-inflammatory macrophages (M1 macrophages). The abnormal activation of PPARγ is an important factor leading to inflammatory osteo-damage related diseases (such as rheumatoid arthritis and osteoporosis). Therefore, regulating the expression of PPARγ in macrophages/osteoclasts is of great significance for the treatment of osteo-damage related diseases.

### ERRα

ERRα belongs to the nuclear receptor superfamily of estrogen-related receptor subtypes. ERRα is related to growth and development and is highly expressed in tissues with high oxidative metabolism (99). In recent years, emerging evidence proves that ERRα can inhibit the differentiation of macrophages and promote the differentiation of osteoclasts (100–106). *In vitro* studies indicated that the expression of ERRα is significantly increased in macrophages activated by LPS or IFNγ stimulation (103, 107). The macrophages of ERRα deficient mice showed excessive inflammatory response and systemic inflammation after immune challenge (103). In-depth studies demonstrated that ERRα negatively regulates inflammation induced by Toll-like receptors (TLRs) through controlling the metabolic process of macrophages and inducing the transcription of TNFα-induced protein 3 (103, 108). Consistently, another study confirmed that the activation of ERRα inhibits the secretion of pro-inflammatory cytokines by activated macrophages (101). Furthermore, ERRα and its auxiliary activator PGC-1β of macrophages can increase the host’s immune defense (102, 105). In conclusion, these data suggest that ERRα is necessary to maintain the homeostasis-function of macrophages.

The study of Bonnelye E et al. showed that ERRα regulates the transport and adhesion of osteoclasts to participate in bone resorption (109, 110). In recent years, the role of ERRα in osteoclasts has been confirmed in ERRα gene knockout mice or animal experiments (100, 111). Specifically, mice with the ERRα gene defect were observed to have osteoporosis due to osteoclasts dysfunction, suggesting that ERRα is an essential regulator of bone resorption and bone remodeling. Furthermore, in osteoclasts, activation of PPARγ with rosiglitazone increased ERRα expression, while deletion of ERRα affected osteoclasts formation (stimulated by rosiglitazone and RANKL). Interestingly, the ERRα gene knockout completely blocked the rosiglitazone-induced activation of mitochondrial biogenesis-activation in osteoclasts. These evidence reveal that ERRα inhibits the formation of macrophages and promotes the formation of osteoclasts.

In general, ERRα is involved in bone resorption and bone remodeling by regulating the functions of osteoclasts and macrophages. Therefore, moderate expression of ERRα is vital for osteo-immune, specifically, ERRα acts as a regulator of the macrophage-osteoclast axis.

### PGC-1β

PGC-1β is widely expressed in tissues such as liver, muscle and brown fat, especially tissues with high oxidizing ability (112, 113). PGC-1β regulates specific tissues by targeting specific transcription factors (such as ERRα and PPARγ). In recent years, emerging evidence demonstrated that PGC-1β has similar effects to PPARγ and ERRα in regulating the functions of macrophages and osteoclasts (114). The activation of M2 macrophages (alternately activated anti-inflammatory macrophages) is accompanied by the induction of oxidative metabolism and the expression of PGC-1β (115, 116). In terms of mechanism, PGC-1β regulates the activation or differentiation of M2 macrophages possibly by promoting the formation of mitochondrial biogenesis and down-regulating the release of pro-inflammatory cytokines (115). Consistent with this, inhibition of mitochondrial oxidative respiration can block the polarization of macrophages to M2 phenotype (117).

In addition to inhibiting the differentiation of pro-inflammatory macrophages, PGC-1β can also induce osteoclasts differentiation. PGC-1β is induced during the process of osteoclasts differentiation caused by cyclic adenosine monophosphate (cAMP) (114, 118). *In vitro* or *in vivo* experiments further demonstrated that down-regulation of PGC-1β inhibits mitochondrial biogenesis and osteoclasts differentiation, and osteoclasts function is severely impaired in PGC-1β-deficient mice (114). Furthermore, Wei et al. found that rosiglitazone highly induces PGC-1β in a PPARγ-dependent manner during osteoclasts differentiation (100).

In conclusion, as the co-agonist of ERRα and PPARγ, PGC-1β promotes osteoclasts differentiation and mitochondrial function and inhibits pro-inflammatory response by regulating the activation of M2 macrophages. Therefore, appropriate activation of PGC-1β is important for the polarization or differentiation of macrophages and osteoclasts.

### NDUFS4

NDUFS4 is an 18 kDa subunit of mitochondrial complex I (CI) located in the inner membrane of mitochondria (119, 120). *In vitro* studies showed that knocking out NDUFS4 in macrophages leads to up-regulation of pro-inflammatory genes, which suggests that the activation of macrophages may be one of the causes of systemic inflammation in NDUFS4-deficient mice (121). Further *in vivo* experiments indicated that NDUFS4 or CI has an autonomous role in regulating the inflammatory response and the activation of M1 (pro-inflammatory macrophages). Interestingly, the expression of pro-inflammatory genes in wild-type macrophages was up-regulated after treatment with rotenone (CI inhibitor). The above data confirm that NDUFS4 or CI inhibits the inflammatory response of macrophages. Contrary to its role in macrophages, the expression of NDUFS4 can promote the formation of osteoclasts. Systemic NDUFS4 knockout or down-regulation of NDUFS4 in hematopoietic cells inhibits the formation of osteoclasts (121). In summary, NDUFS4 inhibits the activation of M1 (pro-inflammatory macrophages) and promotes the differentiation of osteoclasts. Therefore, targeting NDUFS4 to regulate the function of macrophages/osteoclasts may be an effective strategy for the treatment of inflammatory osteo-damage related diseases.

### Maternal VLDLR

Very low-density lipoprotein receptor (VLDLR) belongs to the low-density lipoprotein receptor (LDLR) superfamily and is a transmembrane protein similar to LDLR structurally (122). VLDLR is highly expressed in adipose tissue, heart, and skeletal muscle (tissues that utilize fatty acids), but is not expressed in the intestine and liver (123). Studies indicated that the reconstitution of VLDLR expression in macrophages of VLDLR-deficient mice promotes the occurrence and development of atherosclerosis, which may be due to the accumulation of atherosclerotic lipoproteins caused by the knockout of VLDLR (124, 125). The physical state of the mother has a profound impact on the health of the fetus (126, 127). Maternal-fetal interface studies suggest that VLDLR can induce osteoclasts differentiation and inhibit the inflammatory response of macrophages (128–130). The absence of maternal VLDLR causes the synthesis of phospholipase A2 group 7 (PLA2G7) in macrophages to be blocked, which leads to incomplete milk and the reduction of platelet activating factor acetyl hydrolase (PAFAH) synthesis (128). Platelet activating factor (PAF) is highly expressed in newborns, especially those with inflammatory diseases (131, 132). PAFAH in milk can inhibit the inflammatory response of PAF in newborns and exists in the form of secretion (133, 134). The above evidence reveals that the maternal macrophages VLDLR promotes the production of PAFAH in breast milk to inhibit infant inflammatory response. Interestingly, another study showed that the breast milk of the mother with VLDLR deficiency can inhibit bone resorption due to the obstruction of osteoclasts differentiation, leading to osteoporosis in the offspring (129). In terms of mechanism, the inhibition of osteoclasts differentiation by VLDLR may be due to the promotion of RANKL signaling (129). Collectively, these findings indicate that the maternal VLDLR controls the formation of osteoclasts, which may be achieved by affecting the macrophages.

### Energy Metabolism

**Figure 5:**
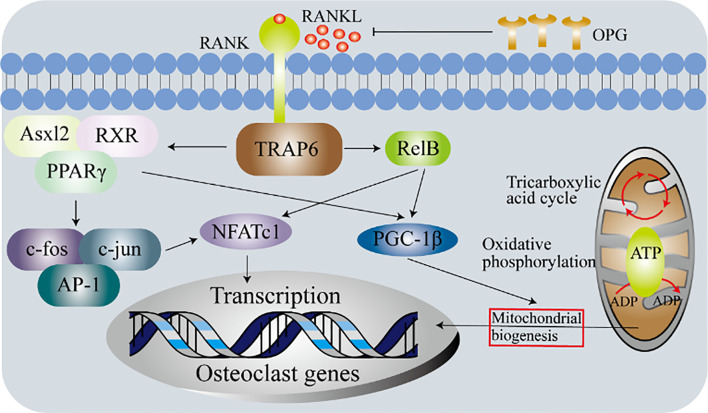
Metabolic determinants of the differentiation osteoclasts. Experiments with primary bone marrow macrophages in Relb or Asxl2-deficient mice show that PGC-1β plays a vital role in the mitochondrial biogenesis of osteoclasts. However, in addition to initiating the expression of osteoclasts genes, c-fos-mediated activation of NFATc1 is also required. These indicate that although mitochondrial biogenesis and osteoclasts differentiation are independent processes, both require the production of functional osteoclasts.

The aforementioned molecules may overlap with energy metabolism in the polarization and/or differentiation of macrophages and osteoclasts. In this section, we focus on the impact of energy metabolism. The role of energy metabolism in osteoclasts is shown in **Figure 5**. Osteoclasts during differentiation (developmental process) can be regarded as a subgroup of macrophages, and energy metabolism is closely related to the polarization of macrophages (135, 136). Metabolic studies on peritoneal macrophages (RAW264.7) and bone marrow-derived macrophages (BMDM) indicated that lysine promotes the activation of M1 and M2, while tyrosine and phenylalanine have opposite effects (137). Proteomics studies revealed that differentiated osteoclasts are rich in lysine degradation proteins, and the biosynthesis of tyrosine, phenylalanine, and tryptophan is promoted. These result in the inhibition of the polarization of macrophages and the enhancement of osteoclasts differentiation (138). The above evidence not only confirms the close connection between macrophages and osteoclasts, but also shows that osteoclasts are a branch of macrophages family. However, so far, whether M1/M2 macrophages can continue to differentiate into osteoclasts is still unclear, which requires new data to support (139). At present, it is generally believed that M2 is alternately activated anti-inflammatory macrophages, while M1 are pro-inflammatory macrophages. Consistent with this, cytokines secreted by M2 such as IL-10 and IL-4 inhibit the expression of nuclear factor of activated T cells-cytoplasmic 1 (NFATc1), which inhibits the formation of osteoclasts. The above evidence proves that M1 macrophages are dependent on glycolysis but M2 macrophages are not dependent on glycolysis. Therefore, based on the above theory, M1 macrophages are likely to be a candidate for the osteoclasts progenitor. It is worth noting that most of the *in vitro* cell experimental models are simple, while the polarization of M1/M2 in physiological environment is much more complicated.

As early as the 1960s, some scholars began to study the role of energy metabolism in osteoclasts formation, but it was only recently that this topic attracted the attention of researchers and was re-discussed. In the process of cell activation and differentiation, the cell will adjust its own energy metabolism according to the actual consumption. The body is a complete concept, and its metabolic disorders affect the function of every cell that makes up the organism (140, 141). Therefore, the damage of bone or cartilage integrity in patients with hyperglycemia can be explained by energy metabolism (142–144). The typical activation of osteoclasts includes several processes: firstly, monocytes/macrophages fuse into multinucleated osteoclasts; then the membrane folds and actin is produced; finally, podosomes acidify cavities and mitochondrial biogenesis increase to allow bone resorption (143). Therefore, the process of converting glucose into adenosine triphosphate (ATP) in mitochondria and the process of glycolysis both play an important role in the differentiation of osteoclasts and bone resorption (143, 145). Furthermore, recent studies suggested that the expression of glucose transporter depends on the level of RANKL, which confirms that osteoclasts differentiation and bone resorption are accompanied by increase in energy metabolism (146–148).

## The Role of the Macrophage-Osteoclast Axis in Osteo-Damage Related Diseases

Under normal physiological conditions, human bone and cartilage are in a dynamic balance of resorption and remodeling, which mainly depends on the functions of osteoclasts and osteoblasts. When the physiological environment changes cause the overproduction of osteoclasts, it leads to osteo-damage related diseases. If the patient does not get timely treatment, it will eventually lead to loss of mobility in severe cases.

### The Macrophage-Osteoclast Axis in Osteosarcoma

**Figure 6:**
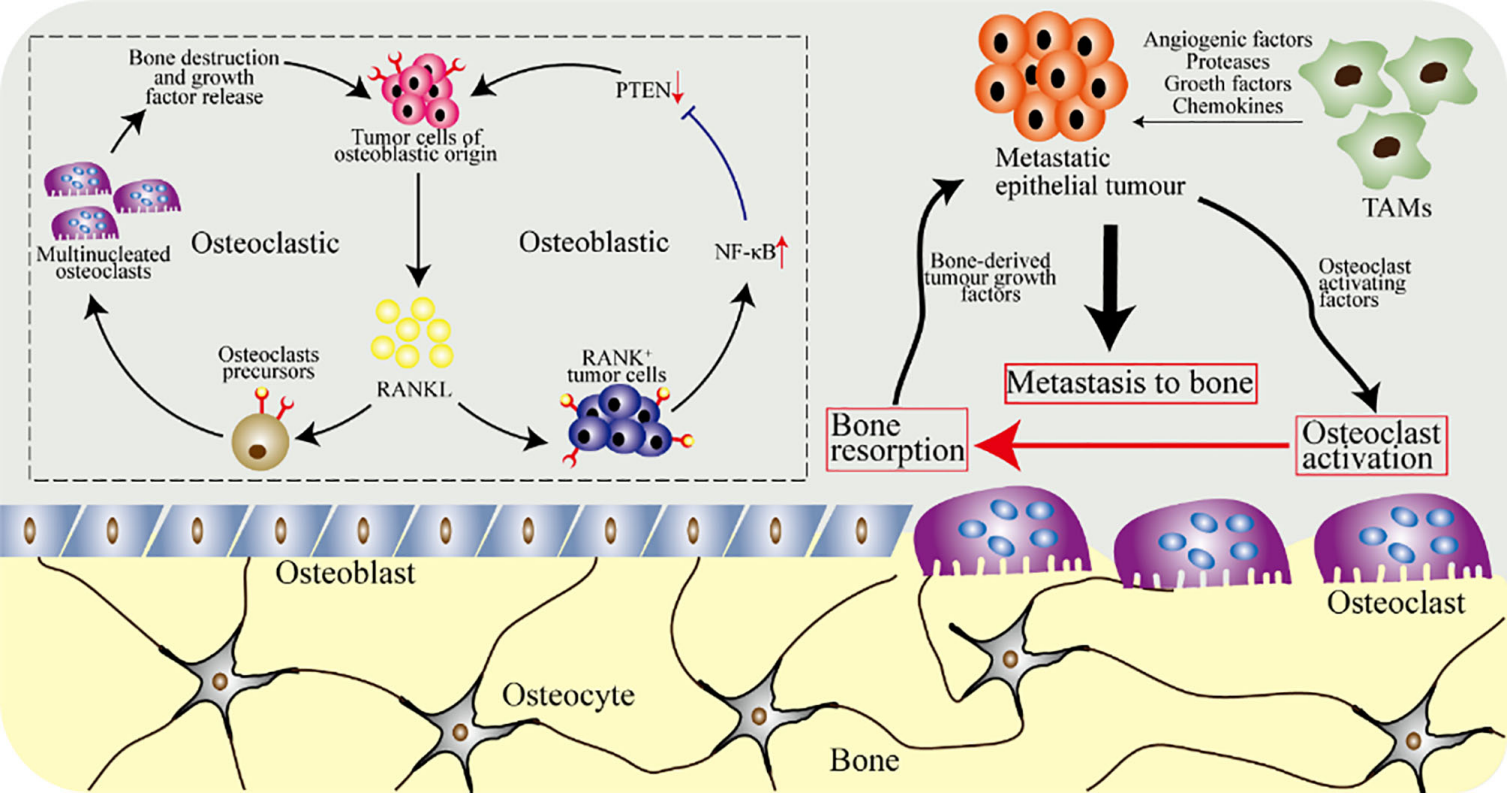
The osteoclasts and macrophages in osteosarcoma. Tumor cells of epithelial origin, such as breast tumor cells, have a tendency to metastasize to bone. Tumor-associated macrophages (TAMs) secrete growth factors and angiogenic factors, and stimulate epithelial tumor cells to secrete proteases and chemokines required for metastasis. Metastatic tumor cells further release cytokines, breaking the balance of osteoclasts and osteoblasts, increasing the activation of osteoclasts and accelerating bone resorption. Furthermore, osteoclasts release cytokines in the bone matrix to further promote the growth of metastatic epithelial tumor cells. The upper left picture shows the cellular and molecular biological effects of local osteosarcoma progression caused by tumor cells and RANKL, which are mediated by osteoblasts and osteoclasts.

The abnormal activation of macrophages contributes to the development of osteosarcoma. On the one hand, infiltrating M2 macrophages (alternatively activated anti-inflammatory macrophages) were observed in metastatic and primary osteosarcoma tissues. On the other hand, Muramyl Tripeptide Phosphoatidyl Ethanolamine (MTP-PE), an immune adjuvant, has made considerable progress in targeting M1 macrophages (proinflammatory macrophages) for the treatment of osteosarcoma. So far, the diametrically opposite roles of M2 and M1 in inflammation have not been clearly elucidated, which may be explained by the plasticity of non-classical patrolling monocytes or M1 and M2. In this section, we focus on the role of macrophages and osteoclasts in osteosarcoma and the potential of targeting these cells to treat the disease (**Figure 6**).

Osteosarcoma is one of the common primary malignant tumors with an incidence rate of about three parts per million. The 5-year survival rate of patients with metastatic osteosarcoma is 15% to 30%, while that of localized osteosarcoma is 80% (149, 150). Immune cells involved in the inflammation of osteosarcoma mainly include CD3^+^T cells, CD14^+^ TAMs (tumor-associated macrophages), and CD68^+^ TAMs. When T cells and TAMs infiltration are evident in osteosarcoma, CD11c^+^ dendritic cells (DC) are also frequently observed. In osteosarcoma, the level of transforming growth factor β (TGF-β) of M2 macrophages affects the survival rate of the patient (151, 152). *In vitro* experiments showed that human osteosarcoma cells secreting osteoclasts-inducing factors were also observed in the presence of TNF-α converting enzyme messenger RNA. Interestingly, RANKL, which plays a central role in osteoclasts and osteosarcoma, belongs to the tumor necrosis factor family. Further studies indicated that the development of human osteosarcoma is accompanied by the recruitment of M2 tumor-associated macrophages, and the growth of osteosarcoma is inhibited after the experimental elimination of macrophages (153, 154). Consistently, CD163^+^ M2 tumor-associated macrophages inhibits the infiltration of T lymphocytes in osteosarcoma, causing osteosarcoma cells to escape the killing of the immune system (151, 155). In osteosarcoma, the macrophages develop and activate under the action of protease, vascular endothelial growth factor (VEGF), and Wnt signaling pathways. Besides, the activation of Wnt signaling pathway can inhibit the formation of osteoclasts by reducing the level of RANKL and promote the differentiation of mesenchymal cells into osteoblasts (156, 157). Furthermore, the activation of the classic Wnt/β-catenin signaling pathway in osteoblasts inhibits the formation of osteoclasts (158).

Targeting macrophages to treat osteosarcoma is an interesting topic, which is a potentially effective strategy. At present, MTP-PE has been approved for the chemotherapy of osteosarcoma and has certain advantages, but the mechanism of MTP-PE in M2 macrophages and M1 macrophages needs to be further clarified. The metastatic or primary and xenogeneic immune infiltration of tumor determine the prognosis of immunotherapy for osteosarcoma. The polarization of macrophages is an alternating and repetitive phenomenon, and clear classification (such as anti-inflammatory phenotype and pro-inflammatory phenotype) helps the treatment of osteosarcoma. Regulating tumor-associated macrophages activation can alleviate bone destruction caused by osteoclasts activation or overproduction.

### The Macrophage-Osteoclast Axis in Rheumatoid Arthritis

**Figure 7:**
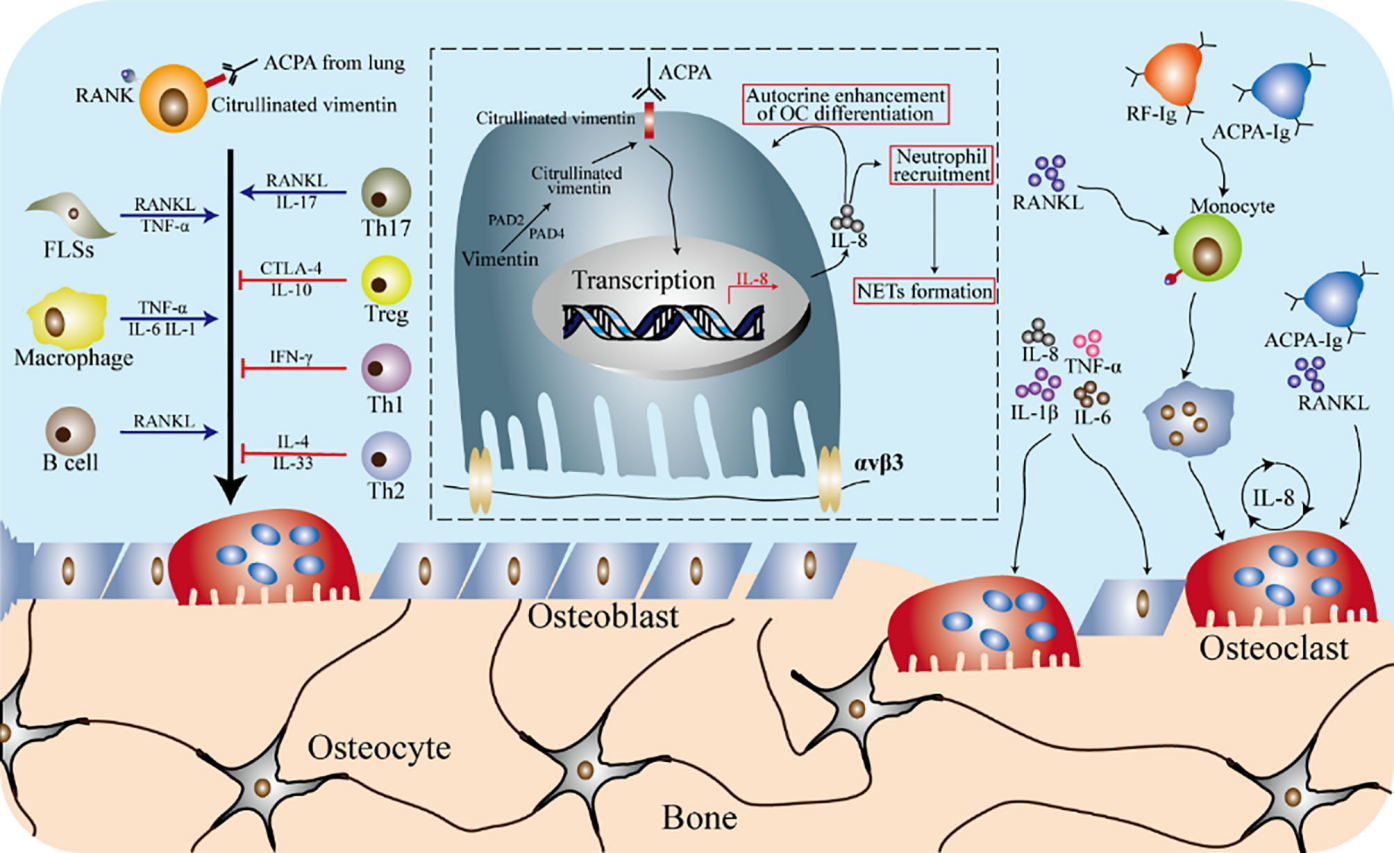
Osteoimmunity in rheumatoid arthritis. The formation of osteoclasts is the result of a variety of immune cells and cytokines in the rheumatoid arthritis (RA) environment. In the inflammatory environment of RA, fibroblast-like synovial cells (FLSs) produce RANKL and TNF-α, and macrophages can produce pro-inflammatory cytokines (such as IL-1β, TNF-α and IL-6), which promote the differentiation of osteoclasts. B cells and T cells participate in the differentiation and activation of osteoclasts by secreting cytokines. ACPA specific to citrulline vimentin can induce the differentiation of osteoclasts precursors and promote the inflammatory response of RA. IL-8 increases the differentiation of osteoclasts precursors in an autocrine manner, and also participates in the recruitment of neutrophils as a chemokine. The NETs formed by the recruited neutrophils play a role in amplifying the inflammation of the synovial tissue, thereby developing RA. Cytokines such as TNF-α, IL-1β, IL6 and IL8 are produced by anti-citrullinated peptide antibodies (ACPA) or rheumatoid factor (RF) autoantibodies attacking the monocytes lineage. These cytokines can not only directly increase the formation of osteoclasts, but also regulate the secretion of RANKL from stromal cells, thereby inducing osteoclasts differentiation. In addition, ACPA promotes the secretion of IL-8 by osteoclasts by affecting osteoclasts.

Rheumatoid arthritis (RA) is an inflammatory autoimmune disease with pathological manifestations of joint pannus formation, joint destruction, and synovial inflammation (159, 160). The role of macrophages and osteoclasts in RA is shown in **Figure 7**. CCR2 and CX3CR1 expressed by circulating monocytes interact with chemokine ligands MCP-1 (CCL2) and Fractalkine (CX3CL1) secreted by fibroblast-like synoviocytes (FLSs) to promote monocytes recruitment to the synovial tissue. In RA, the expression of specific surface antigens of activated monocytes is up-regulated, such as CD16, toll-like receptor (TLR), CD14, adhesion molecule integrin, and HLA-DR. The intermediate monocytes (see section 2.2 for detailed definition) expressing CD14 and CD16 can communicate with other cells and produce pro-inflammatory cytokines (such as IL-1β, IL-6, TNF-α) to promote the occurrence and development of RA. Furthermore, the intermediate monocytes continue to differentiate into M1 macrophages and participates in the promotion of synovial inflammation and joint damage. Emerging evidence suggests that CD14^+^CD16^-^ monocytes can be further differentiated into osteoclasts that causes joint bone destruction. Besides, IL-6, TNF-α, and IL-1β secreted by macrophages recruited to synovial tissue increase the production of osteoclasts. Therefore, in RA, monocytes and macrophages are the circulating precursor of osteoclasts.

Two typical features of RA are joint bone or cartilage damage and synovial inflammation. RA patients with severe clinical symptoms are likely to lose their behavior if they are not treated in time. The normal bone morphology is the result of the dynamic balance between osteoblasts and osteoclasts. To date, osteoclasts are the only primary bone resorptive cell that has been identified, and their abnormal activation or over-formation is responsible for RA. In the joint cavity, synovial macrophages from the circulation can be used as osteoclasts precursors, but there is no evidence that resident synovial macrophages can differentiate into osteoclasts (161). Generally speaking, in the joint inflammation environment of RA, circulating monocytes will continue to transfer to the joint cavity and differentiate into osteoclasts to promote joint inflammation (161, 162). Evidence showed that the monocytes that can differentiate into osteoclasts are CD14 positive but CD16 negative. CD14^+^CD16^-^ monocytes are also the precursor of circulating osteoclasts, rather than a subset of circulating CD16^+^ cell (163, 164). Further data indicated that the expression of RANK receptor is increased on the surface of CD14^+^CD16^-^ monocytes. These results prove that macrophages and osteoclasts are the key factors in the process of RA joint bone or/and cartilage destruction.

### The Macrophages and Osteoclasts in Other Osteo-Damage Related Diseases

The macrophage-osteoclast axis is crucial in osteo-damage related diseases. In addition to the aforementioned osteosarcoma and rheumatoid arthritis, its important role in osteoporosis, osteopetrosis, and osteomyelitis has also been reported. In osteoporosis and other bone loss inflammatory conditions, the macrophage-osteoclast axis plays a vital role. The deletion of the C/ebpα gene in monocytes causes osteopetrosis and inhibits bone resorption in ovariectomized mice, which indicates that the C/ebpα gene is involved in regulating the function of osteoclasts (165). The typical features of osteopetrosis are the obstruction of the formation of osteoclasts and functional defects. A study using RANKL-induced osteoclasts-transgenic osteoporosis as a model revealed that a subset of macrophages is recruited into the bone matrix to participate in the development of the disease (47). The fusion and multinucleation of monocytes/macrophages are indispensable process for the production of osteoclasts, and the activation of these cells determines the balance of bone resorption and bone remodeling (47). Besides, in osteomyelitis, the expression of macrophage-related inflammatory proteins CXCL2 (MIP2α) and CCL3 (MIP1α) is related to the inflammation and bone degradation of osteomyelitis (166). The above evidence confirms the important role of the macrophage-osteoclast axis in osteo-damage related diseases.

## Outlook

Osteoclasts are the only cell known to have bone resorption capacity, which is formed by the differentiation of macrophages or bone marrow precursor. Osteoimmunity is an interdisciplinary field, which mainly involves immunology and osteo-related knowledge. The immune cells involved are macrophages, T cells, B cells, and dendritic cells, and osteo-related cells include osteoclasts and osteoblasts. The macrophage-osteoclast axis plays an essential role in osteoimmunity, regulating the balance of bone remodeling and bone resorption. In addition to the macrophage-osteoclast axis described in this article in osteosarcoma, RA, and other osteo-damage related diseases, the macrophage-osteoclast axis also mediates tumor bone metastasis (167). However, this paper mainly discusses the macrophage-osteoclast axis and osteoimmunity, so it is not discussed. It is worth noting that the current research on macrophages and osteoclasts is mainly *in vitro* cell experiments, and there are still few *in vivo* studies demonstrating that circulation classical monocytes are the osteoclasts precursors in experimental inflammatory arthritis (168). As we all know, the influencing mechanism of macrophages and osteoclasts differentiation or/and activation *in vivo* is very complicated, so simple *in vitro* experiments cannot reflect the essence. Therefore, in-depth *in vivo* studies are necessary to clarify the role of the macrophage-osteoclast axis in osteoimmunity. Under physiological conditions, the determinants of the differentiation of macrophages and osteoclasts are very complex. This article only introduces several molecules that typically affect the differentiation of macrophages and osteoclasts in recent years. So, the molecules that have been discovered but are not typical were not introduced in this article. For example, miR-155 expression precedes and overrides the activation of the osteoclasts transcriptional program, provides the means for coherent macrophages differentiation, even in the presence of osteoclastogenic signals (169). Based on these findings, we propose that miRNA may provide a general mechanism for the unequivocal commitment underlying stem cell differentiation. Furthermore, the process of the differentiation of macrophages subtypes (M1 and M2) into osteoclasts is still unclear. When does M1 differentiate into osteoclasts, and when does M2 differentiate into osteoclasts, in what proportion, and by what factors? These are the urgent problems to be solved when elucidating the role of the macrophage-osteoclast axis in osteoimmunity. In the treatment of osteo-damage related diseases, there is still no therapy for the macrophage-osteoclast axis. Targeting tissue-specific macrophages and inhibiting or promoting differentiation into osteoclasts may be an effective means to regulate bone mass in pathological environments.

## Author Contributions

YY, FR, YQ, and MZ drafted the manuscript. YQ and FR assisted in reviewing literature. CZ, YQY, and FW modified the manuscript. YY and MZ reviewed and edited the final manuscript. XC revised the manuscript. All authors contributed to the article and approved the submitted version.

## Funding

This study was supported by National Natural Science Foundation of China (Grant Nos. 82003761 and 81873838) and Zhejiang Provincial Natural Science Foundation of China (Grant No. LY16H160025).

## Conflict of Interest

The authors declare that the research was conducted in the absence of any commercial or financial relationships that could be constructed as a potential conflict of interest.

## References

[1] Epelman S Lavine KJ Randolph GJ. Origin and functions of tissue macrophages. Immunity (2014) 41(1):21–35. 10.1016/j.immuni.2014.06.013 25035951 PMC4470379

[2] Arron JR Choi Y. Bone versus immune system. Nature (2000) 408(6812):535–6. 10.1038/35046196 11117729

[3] Weitzmann MN Ofotokun I. Physiological and pathophysiological bone turnover - role of the immune system. Nat Rev Endocrinol (2016) 12(9):518–32. 10.1038/nrendo.2016.91 PMC5857945 27312863

[4] Takayanagi H Ogasawara K Hida S Chiba T Murata S Sato K. T-cell-mediated regulation of osteoclastogenesis by signalling cross-talk between RANKL and IFN-gamma. Nature (2000) 408(6812):600–5. 10.1038/35046102 11117749

[5] Fan F Shi P Liu M Chen H Tu M Lu W. Lactoferrin preserves bone homeostasis by regulating the RANKL/RANK/OPG pathway of osteoimmunology. Food Funct (2018) 9(5):2653–60. 10.1039/c8fo00303c 29666861

[6] Anderson DM Maraskovsky E Billingsley WL Dougall WC Tometsko ME Roux ER. A homologue of the TNF receptor and its ligand enhance T-cell growth and dendritic-cell function. Nature (1997) 390(6656):175–9. 10.1038/36593 9367155

[7] Wu L Su Y Lin F Zhu S Wang J Hou Y. MicroRNA-21 promotes orthodontic tooth movement by modulating the RANKL/OPG balance in T cells. Oral Dis (2020) 26(2):370–80. 10.1111/odi.13239 31738470

[8] Kim N Odgren PR Kim DK Marks SC Jr Choi Y. Diverse roles of the tumor necrosis factor family member TRANCE in skeletal physiology revealed by TRANCE deficiency and partial rescue by a lymphocyte-expressed TRANCE transgene. Proc Natl Acad Sci U S A (2000) 97(20):10905–10. 10.1073/pnas.200294797 PMC27122 10984520

[9] Takayanagi H. Osteoimmunology: shared mechanisms and crosstalk between the immune and bone systems. Nat Rev Immunol (2007) 7(4):292–304. 10.1038/nri2062 17380158

[10] Walsh MC Kim N Kadono Y Rho J Lee SY Lorenzo J. Osteoimmunology: interplay between the immune system and bone metabolism. Annu Rev Immunol (2006) 24:33–63. 10.1146/annurev.immunol.24.021605.090646 16551243

[11] Madel MB Ibanez L Wakkach A de Vries TJ Teti A Apparailly F. Immune Function and Diversity of Osteoclasts in Normal and Pathological Conditions. Front Immunol (2019) 10:1408. 10.3389/fimmu.2019.01408 31275328 PMC6594198

[12] Goldring SR. Pathogenesis of bone and cartilage destruction in rheumatoid arthritis. Rheumatol (Oxford) (2003) 42(Suppl 2):ii11–6. 10.1093/rheumatology/keg327 12817090

[13] Walsh NC Gravallese EM. Bone loss in inflammatory arthritis: mechanisms and treatment strategies. Curr Opin Rheumatol (2004) 16(4):419–27. 10.1097/01.bor.0000127824.42507.68 15201606

[14] Kim TH Yang K Kim M Kim HS Kang JL. Apoptosis inhibitor of macrophage (AIM) contributes to IL-10-induced anti-inflammatory response through inhibition of inflammasome activation. Cell Death Dis (2021) 12(1):19. 10.1038/s41419-020-03332-w 33414479 PMC7791024

[15] Nakashima T Hayashi M Takayanagi H. New insights into osteoclastogenic signaling mechanisms. Trends Endocrinol Metab (2012) 23(11):582–90. 10.1016/j.tem.2012.05.005 22705116

[16] Lorenzo J Horowitz M Choi Y. Osteoimmunology: interactions of the bone and immune system. Endocr Rev (2008) 29(4):403–40. 10.1210/er.2007-0038 PMC2528852 18451259

[17] Drouin A Wallbillich N Theberge M Liu S Katz J Bellovoda K. Impact of Zika virus on the human type I interferon osteoimmune response. Cytokine (2021) 137:155342. 10.1016/j.cyto.2020.155342 33130337

[18] Takayanagi H. RANKL as the master regulator of osteoclast differentiation. J Bone Miner Metab (2021) 39(1):13–18. 10.1007/s00774-020-01191-1 33385253

[19] Parfitt AM. High bone turnover is intrinsically harmful: two paths to a similar conclusion. The Parfitt view. J Bone Miner Res (2002) 17(8):1558–9; author reply 1560. 10.1359/jbmr.2002.17.8.1558 12162510

[20] Baron R Kneissel M. WNT signaling in bone homeostasis and disease: from human mutations to treatments. Nat Med (2013) 19(2):179–92. 10.1038/nm.3074 23389618

[21] Teitelbaum SL. Bone resorption by osteoclasts. Science (2000) 289(5484):1504–8. 10.1126/science.289.5484.1504 10968780

[22] Varol C Mildner A Jung S. Macrophages: development and tissue specialization. Annu Rev Immunol (2015) 33:643–75. 10.1146/annurev-immunol-032414-112220 25861979

[23] Demirdelen S Mannes PZ Aral AM Haddad J Leers SA Gomez D. Divergence of acetate uptake in proinflammatory and inflammation-resolving macrophages: implications for imaging atherosclerosis. J Nucl Cardiol (2021). 10.1007/s12350-020-02479-5 PMC8935477 33420659

[24] Chang MK Raggatt LJ Alexander KA Kuliwaba JS Fazzalari NL Schroder K. Osteal tissue macrophages are intercalated throughout human and mouse bone lining tissues and regulate osteoblast function in vitro and in vivo. J Immunol (2008) 181(2):1232–44. 10.4049/jimmunol.181.2.1232 18606677

[25] Sinder BP Pettit AR McCauley LK. Macrophages: Their Emerging Roles in Bone. J Bone Miner Res (2015) 30(12):2140–9. 10.1002/jbmr.2735 PMC4876707 26531055

[26] Gordon S. Elie Metchnikoff: father of natural immunity. Eur J Immunol (2008) 38(12):3257–64. 10.1002/eji.200838855 19039772

[27] Ginhoux F Guilliams M. Tissue-Resident Macrophage Ontogeny and Homeostasis. Immunity (2016) 44(3):439–49. 10.1016/j.immuni.2016.02.024 26982352

[28] Guilliams M Mildner A Yona S. Developmental and Functional Heterogeneity of Monocytes. Immunity (2018) 49(4):595–613. 10.1016/j.immuni.2018.10.005 30332628

[29] Guilliams M Ginhoux F Jakubzick C Naik SH Onai N Schraml BU. Dendritic cells, monocytes and macrophages: a unified nomenclature based on ontogeny. Nat Rev Immunol (2014) 14(8):571–8. 10.1038/nri3712 PMC4638219 25033907

[30] Walker DG. Bone resorption restored in osteopetrotic mice by transplants of normal bone marrow and spleen cells. Science (1975) 190(4216):784–5. 10.1126/science.1105786 1105786

[31] Walker DG. Spleen cells transmit osteopetrosis in mice. Science (1975) 190(4216):785–7. 10.1126/science.1198094 1198094

[32] Udagawa N Takahashi N Akatsu T Tanaka H Sasaki T Nishihara T. Origin of osteoclasts: mature monocytes and macrophages are capable of differentiating into osteoclasts under a suitable microenvironment prepared by bone marrow-derived stromal cells. Proc Natl Acad Sci U S A (1990) 87(18):7260–4. 10.1073/pnas.87.18.7260 PMC54723 2169622

[33] Li L Jiang X Teng S Zhang L Teng L Wang D. Calf thymus polypeptide improved hematopoiesis via regulating colony-stimulating factors in BALB/c mice with hematopoietic dysfunction. Int J Biol Macromol (2020) 156:204–16. 10.1016/j.ijbiomac.2020.03.041 32156537

[34] Nikolic T de Bruijn MF Lutz MB Leenen PJ. Developmental stages of myeloid dendritic cells in mouse bone marrow. Int Immunol (2003) 15(4):515–24. 10.1093/intimm/dxg050 12663681

[35] de Bruijn MF Slieker WA van der Loo JC Voerman JS van Ewijk W Leenen PJ. Distinct mouse bone marrow macrophage precursors identified by differential expression of ER-MP12 and ER-MP20 antigens. Eur J Immunol (1994) 24(10):2279–84. 10.1002/eji.1830241003 7925556

[36] de Vries TJ Schoenmaker T Hooibrink B Leenen PJ Everts V. Myeloid blasts are the mouse bone marrow cells prone to differentiate into osteoclasts. J Leukoc Biol (2009) 85(6):919–27. 10.1189/jlb.0708402 19304896

[37] Baeck C Wei X Bartneck M Fech V Heymann F Gassler N. Pharmacological inhibition of the chemokine C-C motif chemokine ligand 2 (monocyte chemoattractant protein 1) accelerates liver fibrosis regression by suppressing Ly-6C(+) macrophage infiltration in mice. Hepatology (2014) 59(3):1060–72. 10.1002/hep.26783 24481979

[38] Speziani C Rivollier A Gallois A Coury F Mazzorana M Azocar O. Murine dendritic cell transdifferentiation into osteoclasts is differentially regulated by innate and adaptive cytokines. Eur J Immunol (2007) 37(3):747–57. 10.1002/eji.200636534 17304626

[39] Miyamoto T Ohneda O Arai F Iwamoto K Okada S Takagi K. Bifurcation of osteoclasts and dendritic cells from common progenitors. Blood (2001) 98(8):2544–54. 10.1182/blood.v98.8.2544 11588053

[40] Ziegler-Heitbrock L Ancuta P Crowe S Dalod M Grau V Hart DN. Nomenclature of monocytes and dendritic cells in blood. Blood (2010) 116(16):e74–80. 10.1182/blood-2010-02-258558 20628149

[41] Xue J Xu L Zhu H Bai M Li X Zhao Z. CD14(+)CD16(-) monocytes are the main precursors of osteoclasts in rheumatoid arthritis via expressing Tyro3TK. Arthritis Res Ther (2020) 22(1):221. 10.1186/s13075-020-02308-7 32958023 PMC7507256

[42] Liu W Zhang Y Zhu W Ma C Ruan J Long H. Sinomenine Inhibits the Progression of Rheumatoid Arthritis by Regulating the Secretion of Inflammatory Cytokines and Monocyte/Macrophage Subsets. Front Immunol (2018) 9:2228. 10.3389/fimmu.2018.02228 30319663 PMC6168735

[43] Sprangers S Schoenmaker T Cao Y Everts V de Vries TJ. Different Blood-Borne Human Osteoclast Precursors Respond in Distinct Ways to IL-17A. J Cell Physiol (2016) 231(6):1249–60. 10.1002/jcp.25220 26491867

[44] Wypasek E Padjas A Szymanska M Plens K Siedlar M Undas A. Non-classical and intermediate monocytes in patients following venous thromboembolism: Links with inflammation. Adv Clin Exp Med (2019) 28(1):51–8. 10.17219/acem/76262 30088349

[45] Ravenhill BJ Soday L Houghton J Antrobus R Weekes MP. Comprehensive cell surface proteomics defines markers of classical, intermediate and non-classical monocytes. Sci Rep (2020) 10(1):4560. 10.1038/s41598-020-61356-w 32165698 PMC7067879

[46] Cren M Nziza N Carbasse A Mahe P Dufourcq-Lopez E Delpont M. Differential Accumulation and Activation of Monocyte and Dendritic Cell Subsets in Inflamed Synovial Fluid Discriminates Between Juvenile Idiopathic Arthritis and Septic Arthritis. Front Immunol (2020) 11:1716. 10.3389/fimmu.2020.01716 32849606 PMC7411147

[47] Gambari L Grassi F Roseti L Grigolo B Desando G. Learning from Monocyte-Macrophage Fusion and Multinucleation: Potential Therapeutic Targets for Osteoporosis and Rheumatoid Arthritis. Int J Mol Sci (2020) 21(17):6001. 10.3390/ijms21176001 PMC7504439 32825443

[48] Yagi M Miyamoto T Toyama Y Suda T. Role of DC-STAMP in cellular fusion of osteoclasts and macrophage giant cells. J Bone Miner Metab (2006) 24(5):355–8. 10.1007/s00774-006-0697-9 16937266

[49] Cui W Ke JZ Zhang Q Ke HZ Chalouni C Vignery A. The intracellular domain of CD44 promotes the fusion of macrophages. Blood (2006) 107(2):796–805. 10.1182/blood-2005-05-1902 16195325 PMC1473173

[50] Takeda Y Tachibana I Miyado K Kobayashi M Miyazaki T Funakoshi T. Tetraspanins CD9 and CD81 function to prevent the fusion of mononuclear phagocytes. J Cell Biol (2003) 161(5):945–56. 10.1083/jcb.200212031 PMC2172976 12796480

[51] Eleveld-Trancikova D Triantis V Moulin V Looman MW Wijers M Fransen JA. The dendritic cell-derived protein DC-STAMP is highly conserved and localizes to the endoplasmic reticulum. J Leukoc Biol (2005) 77(3):337–43. 10.1189/jlb.0804441 15601667

[52] Il’in DA Shkurupy VA. In Vitro Analysis of the Expression of CD11, CD29, CD36, and DC-STAMP Molecules during the Formation of Multinuclear Macrophages in BCG-Infected Mice. Bull Exp Biol Med (2019) 167(5):653–5. 10.1007/s10517-019-04591-0 31641985

[53] Miyamoto H Suzuki T Miyauchi Y Iwasaki R Kobayashi T Sato Y. Osteoclast stimulatory transmembrane protein and dendritic cell-specific transmembrane protein cooperatively modulate cell-cell fusion to form osteoclasts and foreign body giant cells. J Bone Miner Res (2012) 27(6):1289–97. 10.1002/jbmr.1575 22337159

[54] Khan UA Hashimi SM Bakr MM Forwood MR Morrison NA. Foreign body giant cells and osteoclasts are TRAP positive, have podosome-belts and both require OC-STAMP for cell fusion. J Cell Biochem (2013) 114(8):1772–8. 10.1002/jcb.24518 23444125

[55] Parthasarathy V Martin F Higginbottom A Murray H Moseley GW Read RC. Distinct roles for tetraspanins CD9, CD63 and CD81 in the formation of multinucleated giant cells. Immunology (2009) 127(2):237–48. 10.1111/j.1365-2567.2008.02945.x PMC2691789 19489128

[56] Jin CH Xia J Rafiq S Huang X Hu Z Zhou X. Modeling anti-CD19 CAR T cell therapy in humanized mice with human immunity and autologous leukemia. EBioMedicine (2019) 39:173–81. 10.1016/j.ebiom.2018.12.013 PMC6354733 30579863

[57] Mordica WJ Woods KM Clem RJ Passarelli AL Chapes SK. Macrophage cell lines use CD81 in cell growth regulation. In Vitro Cell Dev Biol Anim (2009) 45(5-6):213–25. 10.1007/s11626-008-9167-0 PMC3595608 19184252

[58] Sterling H Saginario C Vignery A. CD44 occupancy prevents macrophage multinucleation. J Cell Biol (1998) 143(3):837–47. 10.1083/jcb.143.3.837 PMC2148144 9813101

[59] Gomez KE Wu F Keysar SB Morton JJ Miller B Chimed TS. Cancer Cell CD44 Mediates Macrophage/Monocyte-Driven Regulation of Head and Neck Cancer Stem Cells. Cancer Res (2020) 80(19):4185–98. 10.1158/0008-5472.CAN-20-1079 PMC8146866 32816856

[60] Li Y Zhong G Sun W Zhao C Zhang P Song J. CD44 deficiency inhibits unloading-induced cortical bone loss through downregulation of osteoclast activity. Sci Rep (2015) 5:16124. 10.1038/srep16124 26530337 PMC4632082

[61] Chellaiah MA Ma T. Membrane localization of membrane type 1 matrix metalloproteinase by CD44 regulates the activation of pro-matrix metalloproteinase 9 in osteoclasts. BioMed Res Int (2013) 2013:302392. 10.1155/2013/302392 23984338 PMC3745902

[62] Gonzalo P Arroyo AG. MT1-MMP: A novel component of the macrophage cell fusion machinery. Commun Integr Biol (2010) 3(3):256–9. 10.4161/cib.3.3.11456 PMC2918771 20714408

[63] Shaverdashvili K Wong P Ma J Zhang K Osman I Bedogni B. MT1-MMP modulates melanoma cell dissemination and metastasis through activation of MMP2 and RAC1. Pigment Cell Melanoma Res (2014) 27(2):287–96. 10.1111/pcmr.12201 24387669

[64] Jansen ID Vermeer JA Bloemen V Stap J Everts V. Osteoclast fusion and fission. Calcif Tissue Int (2012) 90(6):515–22. 10.1007/s00223-012-9600-y PMC3349023 22527205

[65] Fang JY Yang Z Han B. Switch of macrophage fusion competency by 3D matrices. Sci Rep (2020) 10(1):10348. 10.1038/s41598-020-67056-9 32587271 PMC7316750

[66] Boissy P Saltel F Bouniol C Jurdic P Machuca-Gayet I. Transcriptional activity of nuclei in multinucleated osteoclasts and its modulation by calcitonin. Endocrinology (2002) 143(5):1913–21. 10.1210/endo.143.5.8813 11956174

[67] Takito J Nakamura M. Heterogeneity and Actin Cytoskeleton in Osteoclast and Macrophage Multinucleation. Int J Mol Sci (2020) 21(18):6629. 10.3390/ijms21186629 PMC7554939 32927783

[68] Pagani F Francucci CM Moro L. Markers of bone turnover: biochemical and clinical perspectives. J Endocrinol Invest (2005) 28(10 Suppl):8–13. 16550716

[69] Chiu WS McManus JF Notini AJ Cassady AI Zajac JD Davey RA. Transgenic mice that express Cre recombinase in osteoclasts. Genesis (2004) 39(3):178–85. 10.1002/gene.20041 15282744

[70] Gao LH Li SS Yue H Zhang ZL. Associations of Serum Cathepsin K and Polymorphisms in CTSK Gene With Bone Mineral Density and Bone Metabolism Markers in Postmenopausal Chinese Women. Front Endocrinol (Lausanne) (2020) 11:48. 10.3389/fendo.2020.00048 32117071 PMC7031211

[71] Reponen P Sahlberg C Munaut C Thesleff I Tryggvason K. High expression of 92-kDa type IV collagenase (gelatinase) in the osteoclast lineage during mouse development. Ann N Y Acad Sci (1994) 732:472–5. 10.1111/j.1749-6632.1994.tb24789.x 7978842

[72] Guo J Zeng X Miao J Liu C Wei F Liu D. MiRNA-218 regulates osteoclast differentiation and inflammation response in periodontitis rats through Mmp9. Cell Microbiol (2019) 21(4):e12979. 10.1111/cmi.12979 30444938

[73] Sugatani T Agapova OA Fang Y Berman AG Wallace JM Malluche HH. Ligand trap of the activin receptor type IIA inhibits osteoclast stimulation of bone remodeling in diabetic mice with chronic kidney disease. Kidney Int (2017) 91(1):86–95. 10.1016/j.kint.2016.07.039 27666759 PMC5530394

[74] Hayman AR. Tartrate-resistant acid phosphatase (TRAP) and the osteoclast/immune cell dichotomy. Autoimmunity (2008) 41(3):218–23. 10.1080/08916930701694667 18365835

[75] Vallee A Vallee JN Lecarpentier Y. PPARgamma agonists: potential treatment for autism spectrum disorder by inhibiting the canonical WNT/beta-catenin pathway. Mol Psychiatry (2019) 24(5):643–52. 10.1038/s41380-018-0131-4 30104725

[76] Moore KJ Rosen ED Fitzgerald ML Randow F Andersson LP Altshuler D. The role of PPAR-gamma in macrophage differentiation and cholesterol uptake. Nat Med (2001) 7(1):41–7. 10.1038/83328 11135614

[77] Tontonoz P Nagy L Alvarez JG Thomazy VA Evans RM. PPARgamma promotes monocyte/macrophage differentiation and uptake of oxidized LDL. Cell (1998) 93(2):241–52. 10.1016/s0092-8674(00)81575-5 9568716

[78] Daniel B Nagy G Czimmerer Z Horvath A Hammers DW Cuaranta-Monroy I. The Nuclear Receptor PPARgamma Controls Progressive Macrophage Polarization as a Ligand-Insensitive Epigenomic Ratchet of Transcriptional Memory. Immunity (2018) 49(4):615–626 e6. 10.1016/j.immuni.2018.09.005 30332629 PMC6197058

[79] Ricote M Li AC Willson TM Kelly CJ Glass CK. The peroxisome proliferator-activated receptor-gamma is a negative regulator of macrophage activation. Nature (1998) 391(6662):79–82. 10.1038/34178 9422508

[80] Heming M Gran S Jauch SL Fischer-Riepe L Russo A Klotz L. Peroxisome Proliferator-Activated Receptor-gamma Modulates the Response of Macrophages to Lipopolysaccharide and Glucocorticoids. Front Immunol (2018) 9:893. 10.3389/fimmu.2018.00893 29867927 PMC5949563

[81] Chawla A Barak Y Nagy L Liao D Tontonoz P Evans RM. PPAR-gamma dependent and independent effects on macrophage-gene expression in lipid metabolism and inflammation. Nat Med (2001) 7(1):48–52. 10.1038/83336 11135615

[82] Schneider C Nobs SP Kurrer M Rehrauer H Thiele C Kopf M. Induction of the nuclear receptor PPAR-gamma by the cytokine GM-CSF is critical for the differentiation of fetal monocytes into alveolar macrophages. Nat Immunol (2014) 15(11):1026–37. 10.1038/ni.3005 25263125

[83] Jiang C Ting AT Seed B. PPAR-gamma agonists inhibit production of monocyte inflammatory cytokines. Nature (1998) 391(6662):82–6. 10.1038/34184 9422509

[84] Wan Y Evans RM. Rosiglitazone activation of PPARgamma suppresses fractalkine signaling. J Mol Endocrinol (2010) 44(2):135–42. 10.1677/JME-09-0090 PMC2805405 19850645

[85] Odegaard JI Ricardo-Gonzalez RR Goforth MH Morel CR Subramanian V Mukundan L. Macrophage-specific PPARgamma controls alternative activation and improves insulin resistance. Nature (2007) 447(7148):1116–20. 10.1038/nature05894 PMC2587297 17515919

[86] Bouhlel MA Derudas B Rigamonti E Dievart R Brozek J Haulon S. PPARgamma activation primes human monocytes into alternative M2 macrophages with anti-inflammatory properties. Cell Metab (2007) 6(2):137–43. 10.1016/j.cmet.2007.06.010 17681149

[87] Wan Y Chong LW Evans RM. PPAR-gamma regulates osteoclastogenesis in mice. Nat Med (2007) 13(12):1496–503. 10.1038/nm1672 18059282

[88] Jin Z Wei W Huynh H Wan Y. HDAC9 Inhibits Osteoclastogenesis via Mutual Suppression of PPARgamma/RANKL Signaling. Mol Endocrinol (2015) 29(5):730–8. 10.1210/me.2014-1365 PMC4415206 25793404

[89] Park KL Oh DG Kim YO Song KS Ahn DW. Rosiglitazone suppresses RANKL-induced NFATc1 autoamplification by disrupting the physical interaction between NFATc1 and PPARgamma. FEBS Open Bio (2018) 8(10):1584–93. 10.1002/2211-5463.12513 PMC6168694 30338210

[90] Grigoriadis AE Wang ZQ Cecchini MG Hofstetter W Felix R Fleisch HA. c-Fos: a key regulator of osteoclast-macrophage lineage determination and bone remodeling. Science (1994) 266(5184):443–8. 10.1126/science.7939685 7939685

[91] Grey A Bolland M Gamble G Wattie D Horne A Davidson J. The peroxisome proliferator-activated receptor-gamma agonist rosiglitazone decreases bone formation and bone mineral density in healthy postmenopausal women: a randomized, controlled trial. J Clin Endocrinol Metab (2007) 92(4):1305–10. 10.1210/jc.2006-2646 17264176

[92] Rzonca SO Suva LJ Gaddy D Montague DC Lecka-Czernik B. Bone is a target for the antidiabetic compound rosiglitazone. Endocrinology (2004) 145(1):401–6. 10.1210/en.2003-0746 PMC1855213 14500573

[93] Sottile V Seuwen K Kneissel M. Enhanced marrow adipogenesis and bone resorption in estrogen-deprived rats treated with the PPARgamma agonist BRL49653 (rosiglitazone). Calcif Tissue Int (2004) 75(4):329–37. 10.1007/s00223-004-0224-8 15549648

[94] Lin S Ke D Lin Y Fu X Yu Y. Puerarin inhibits the migration of osteoclast precursors and osteoclastogenesis by inhibiting MCP-1 production. Biosci Biotechnol Biochem (2020) 84(7):1455–9. 10.1080/09168451.2020.1738912 32154764

[95] Wei W Zeve D Wang X Du Y Tang W Dechow PC. Osteoclast progenitors reside in the peroxisome proliferator-activated receptor gamma-expressing bone marrow cell population. Mol Cell Biol (2011) 31(23):4692–705. 10.1128/MCB.05979-11 PMC3232921 21947280

[96] Akune T Ohba S Kamekura S Yamaguchi M Chung UI Kubota N. PPARgamma insufficiency enhances osteogenesis through osteoblast formation from bone marrow progenitors. J Clin Invest (2004) 113(6):846–55. 10.1172/JCI19900 PMC362117 15067317

[97] Lazarenko OP Rzonca SO Hogue WR Swain FL Suva LJ Lecka-Czernik B. Rosiglitazone induces decreases in bone mass and strength that are reminiscent of aged bone. Endocrinology (2007) 148(6):2669–80. 10.1210/en.2006-1587 PMC2084459 17332064

[98] Wan Y. PPARgamma in bone homeostasis. Trends Endocrinol Metab (2010) 21(12):722–8. 10.1016/j.tem.2010.08.006 20863714

[99] Liu SL Wu XS Li FN Yao WY Wu ZY Dong P. ERRalpha promotes pancreatic cancer progression by enhancing the transcription of PAI1 and activating the MEK/ERK pathway. Am J Cancer Res (2020) 10(11):3622–43. 10.21203/rs.3.rs-33052/v1 PMC7716152 33294258

[100] Wei W Wang X Yang M Smith LC Dechow PC Sonoda J. PGC1beta mediates PPARgamma activation of osteoclastogenesis and rosiglitazone-induced bone loss. Cell Metab (2010) 11(6):503–16. 10.1016/j.cmet.2010.04.015 PMC3521515 20519122

[101] Wei W Schwaid AG Wang X Wang X Chen S Chu Q. Ligand Activation of ERRalpha by Cholesterol Mediates Statin and Bisphosphonate Effects. Cell Metab (2016) 23(3):479–91. 10.1016/j.cmet.2015.12.010 PMC4785078 26777690

[102] Sonoda J Laganiere J Mehl IR Barish GD Chong LW Li X. Nuclear receptor ERR alpha and coactivator PGC-1 beta are effectors of IFN-gamma-induced host defense. Genes Dev (2007) 21(15):1909–20. 10.1101/gad.1553007 PMC1935029 17671090

[103] Yuk JM Kim TS Kim SY Lee HM Han J Dufour CR. Orphan Nuclear Receptor ERRalpha Controls Macrophage Metabolic Signaling and A20 Expression to Negatively Regulate TLR-Induced Inflammation. Immunity (2015) 43(1):80–91. 10.1016/j.immuni.2015.07.003 26200012

[104] He X Ma S Tian Y Wei C Zhu Y Li F. ERRalpha negatively regulates type I interferon induction by inhibiting TBK1-IRF3 interaction. PLoS Pathog (2017) 13(6):e1006347. 10.1371/journal.ppat.1006347 28591144 PMC5476288

[105] Kim SY Yang CS Lee HM Kim JK Kim YS Kim YR. ESRRA (estrogen-related receptor alpha) is a key coordinator of transcriptional and post-translational activation of autophagy to promote innate host defense. Autophagy (2018) 14(1):152–68. 10.1080/15548627.2017.1339001 PMC5846564 28841353

[106] Bae S Lee MJ Mun SH Giannopoulou EG Yong-Gonzalez V Cross JR. MYC-dependent oxidative metabolism regulates osteoclastogenesis via nuclear receptor ERRalpha. J Clin Invest (2017) 127(7):2555–68. 10.1172/JCI89935 PMC5490751 28530645

[107] Barish GD Downes M Alaynick WA Yu RT Ocampo CB Bookout AL. A Nuclear Receptor Atlas: macrophage activation. Mol Endocrinol (2005) 19(10):2466–77. 10.1210/me.2004-0529 16051664

[108] Song W Li D Tao L Luo Q Chen L. Solute carrier transporters: the metabolic gatekeepers of immune cells. Acta Pharm Sin B (2020) 10(1):61–78. 10.1016/j.apsb.2019.12.006 31993307 PMC6977534

[109] Bonnelye E Saltel F Chabadel A Zirngibl RA Aubin JE Jurdic P. Involvement of the orphan nuclear estrogen receptor-related receptor alpha in osteoclast adhesion and transmigration. J Mol Endocrinol (2010) 45(6):365–77. 10.1677/JME-10-0024 PMC2990392 20841427

[110] Yang D Wan Y. Molecular determinants for the polarization of macrophage and osteoclast. Semin Immunopathol (2019) 41(5):551–63. 10.1007/s00281-019-00754-3 PMC6815265 31506868

[111] Zheng ZG Cheng HM Zhou YP Zhu ST Thu PM Li HJ. Dual targeting of SREBP2 and ERRalpha by carnosic acid suppresses RANKL-mediated osteoclastogenesis and prevents ovariectomy-induced bone loss. Cell Death Differ (2020) 27(7):2048–65. 10.1038/s41418-019-0484-5 PMC7308277 31907393

[112] Lin J Puigserver P Donovan J Tarr P Spiegelman BM. Peroxisome proliferator-activated receptor gamma coactivator 1beta (PGC-1beta ), a novel PGC-1-related transcription coactivator associated with host cell factor. J Biol Chem (2002) 277(3):1645–8. 10.1074/jbc.C100631200 11733490

[113] Nakai S Oyabu M Hatazawa Y Akashi S Kitamura T Miura S. FOXO1 suppresses PGC-1beta gene expression in skeletal muscles. FEBS Open Bio (2020) 10(7):1373–88. 10.1002/2211-5463.12898 PMC7327905 32433820

[114] Ishii KA Fumoto T Iwai K Takeshita S Ito M Shimohata N. Coordination of PGC-1beta and iron uptake in mitochondrial biogenesis and osteoclast activation. Nat Med (2009) 15(3):259–66. 10.1038/nm.1910 19252502

[115] Vats D Mukundan L Odegaard JI Zhang L Smith KL Morel CR. Oxidative metabolism and PGC-1beta attenuate macrophage-mediated inflammation. Cell Metab (2006) 4(1):13–24. 10.1016/j.cmet.2006.05.011 16814729 PMC1904486

[116] Galvan-Pena S O’Neill LA. Metabolic reprograming in macrophage polarization. Front Immunol (2014) 5:420. 10.3389/fimmu.2014.00420 25228902 PMC4151090

[117] Van den Bossche J Baardman J Otto NA van der Velden S Neele AE van den Berg SM. Mitochondrial Dysfunction Prevents Repolarization of Inflammatory Macrophages. Cell Rep (2016) 17(3):684–96. 10.1016/j.celrep.2016.09.008 27732846

[118] Ma JD Jing J Wang JW Mo YQ Li QH Lin JZ. Activation of the Peroxisome Proliferator-Activated Receptor gamma Coactivator 1beta/NFATc1 Pathway in Circulating Osteoclast Precursors Associated With Bone Destruction in Rheumatoid Arthritis. Arthritis Rheumatol (2019) 71(8):1252–64. 10.1002/art.40868 PMC6771785 30802366

[119] Johnson SC Kayser EB Bornstein R Stokes J Bitto A Park KY. Regional metabolic signatures in the Ndufs4(KO) mouse brain implicate defective glutamate/alpha-ketoglutarate metabolism in mitochondrial disease. Mol Genet Metab (2020) 130(2):118–32. 10.1016/j.ymgme.2020.03.007 PMC7272141 32331968

[120] Adjobo-Hermans MJW de Haas R Willems P Wojtala A van Emst-de Vries SE Wagenaars JA. NDUFS4 deletion triggers loss of NDUFA12 in Ndufs4(-/-) mice and Leigh syndrome patients: A stabilizing role for NDUFAF2. Biochim Biophys Acta Bioenerg (2020) 1861(8):148213. 10.1016/j.bbabio.2020.148213 32335026

[121] Jin Z Wei W Yang M Du Y Wan Y. Mitochondrial complex I activity suppresses inflammation and enhances bone resorption by shifting macrophage-osteoclast polarization. Cell Metab (2014) 20(3):483–98. 10.1016/j.cmet.2014.07.011 PMC4156549 25130399

[122] Hirota Y Nakajima K. VLDLR is not essential for reelin-induced neuronal aggregation but suppresses neuronal invasion into the marginal zone. Development (2020) 147(12):dev189936. 10.1242/dev.189936 32540847

[123] Zarei M Barroso E Palomer X Escola-Gil JC Cedo L Wahli W. Pharmacological PPARbeta/delta activation upregulates VLDLR in hepatocytes. Clin Investig Arterioscler (2019) 31(3):111–8. 10.1016/j.arteri.2019.01.004 30987865

[124] Eck MV Oost J Goudriaan JR Hoekstra M Hildebrand RB Bos IS. Role of the macrophage very-low-density lipoprotein receptor in atherosclerotic lesion development. Atherosclerosis (2005) 183(2):230–7. 10.1016/j.atherosclerosis.2005.03.045 15979629

[125] Shin KC Hwang I Choe SS Park J Ji Y Kim JI. Macrophage VLDLR mediates obesity-induced insulin resistance with adipose tissue inflammation. Nat Commun (2017) 8(1):1087. 10.1038/s41467-017-01232-w 29057873 PMC5651811

[126] Yao Y Cai X Chen C Fang H Zhao Y Fei W. The Role of Microbiomes in Pregnant Women and Offspring: Research Progress of Recent Years. Front Pharmacol (2020) 11:643. 10.3389/fphar.2020.00643 32457628 PMC7225329

[127] Yao Y Cai X Fei W Ren F Wang F Luan X. Regulating Gut Microbiome: Therapeutic Strategy for Rheumatoid Arthritis During Pregnancy and Lactation. Front Pharmacol (2020) 11:594042. 10.3389/fphar.2020.594042 33343364 PMC7748111

[128] Du Y Yang M Wei W Huynh HD Herz J Saghatelian A. Macrophage VLDL receptor promotes PAFAH secretion in mother’s milk and suppresses systemic inflammation in nursing neonates. Nat Commun (2012) 3:1008. 10.1038/ncomms2011 22910354 PMC3520613

[129] Huynh H Wei W Wan Y. mTOR Inhibition Subdues Milk Disorder Caused by Maternal VLDLR Loss. Cell Rep (2017) 19(10):2014–25. 10.1016/j.celrep.2017.05.037 PMC5526096 28591574

[130] Yang D Huynh H Wan Y. Milk lipid regulation at the maternal-offspring interface. Semin Cell Dev Biol (2018) 81:141–8. 10.1016/j.semcdb.2017.10.012 PMC5916746 29051053

[131] Caplan MS Sun XM Hseuh W Hageman JR. Role of platelet activating factor and tumor necrosis factor-alpha in neonatal necrotizing enterocolitis. J Pediatr (1990) 116(6):960–4. 10.1016/s0022-3476(05)80661-4 2348301

[132] Walker A. Breast milk as the gold standard for protective nutrients. J Pediatr (2010) 156(2 Suppl):S3–7. 10.1016/j.jpeds.2009.11.021 20105662

[133] Furukawa M Narahara H Yasuda K Johnston JM. Presence of platelet-activating factor-acetylhydrolase in milk. J Lipid Res (1993) 34(9):1603–9. 10.1016/S0022-2275(20)36953-4 8228643

[134] Moya FR Eguchi H Zhao B Furukawa M Sfeir J Osorio M. Platelet-activating factor acetylhydrolase in term and preterm human milk: a preliminary report. J Pediatr Gastroenterol Nutr (1994) 19(2):236–9. 10.1097/00005176-199408000-00015 7815247

[135] Horwood NJ. Macrophage Polarization and Bone Formation: A review. Clin Rev Allergy Immunol (2016) 51(1):79–86. 10.1007/s12016-015-8519-2 26498771

[136] Alexander RK Liou YH Knudsen NH Starost KA Xu C Hyde AL. Bmal1 integrates mitochondrial metabolism and macrophage activation. Elife (2020) 9:e54090. 10.7554/eLife.54090 32396064 PMC7259948

[137] Bordbar A Mo ML Nakayasu ES Schrimpe-Rutledge AC Kim YM Metz TO. Model-driven multi-omic data analysis elucidates metabolic immunomodulators of macrophage activation. Mol Syst Biol (2012) 8:558. 10.1038/msb.2012.21 22735334 PMC3397418

[138] An E Narayanan M Manes NP Nita-Lazar A. Characterization of functional reprogramming during osteoclast development using quantitative proteomics and mRNA profiling. Mol Cell Proteomics (2014) 13(10):2687–704. 10.1074/mcp.M113.034371 PMC4188996 25044017

[139] Lampiasi N Russo R Zito F. The Alternative Faces of Macrophage Generate Osteoclasts. BioMed Res Int (2016) 2016:9089610. 10.1155/2016/9089610 26977415 PMC4761668

[140] Yao Y Cai X Fei W Ye Y Zhao M Zheng C. The role of short-chain fatty acids in immunity, inflammation and metabolism. Crit Rev Food Sci Nutr (2020), 1–12. 10.1080/10408398.2020.1854675 33261516

[141] Maria Louis J Agarwal A Aduri R Talukdar I. Global Analysis of RNA-Protein Interactions in TNF-alpha Induced Alternative Splicing in Metabolic Disorders. FEBS Lett (2021) 595(4):476–90. 10.1002/1873-3468.14029 33417721

[142] Fong JE Le Nihouannen D Tiedemann K Sadvakassova G Barralet JE Komarova SV. Moderate excess of pyruvate augments osteoclastogenesis. Biol Open (2013) 2(4):387–95. 10.1242/bio.20133269 PMC3625867 23616923

[143] Motyl KJ Guntur AR Carvalho AL Rosen CJ. Energy Metabolism of Bone. Toxicol Pathol (2017) 45(7):887–93. 10.1177/0192623317737065 PMC5777524 29096593

[144] Cipriani C Colangelo L Santori R Renella M Mastrantonio M Minisola S. The Interplay Between Bone and Glucose Metabolism. Front Endocrinol (Lausanne) (2020) 11:122. 10.3389/fendo.2020.00122 32265831 PMC7105593

[145] Karner CM Long F. Glucose metabolism in bone. Bone (2018) 115:2–7. 10.1016/j.bone.2017.08.008 28843700 PMC6030501

[146] Ahn H Lee K Kim JM Kwon SH Lee SH Lee SY. Accelerated Lactate Dehydrogenase Activity Potentiates Osteoclastogenesis via NFATc1 Signaling. PLoS One (2016) 11(4):e0153886. 10.1371/journal.pone.0153886 27077737 PMC4831772

[147] Kim JM Jeong D Kang HK Jung SY Kang SS Min BM. Osteoclast precursors display dynamic metabolic shifts toward accelerated glucose metabolism at an early stage of RANKL-stimulated osteoclast differentiation. Cell Physiol Biochem (2007) 20(6):935–46. 10.1159/000110454 17982276

[148] Indo Y Takeshita S Ishii KA Hoshii T Aburatani H Hirao A. Metabolic regulation of osteoclast differentiation and function. J Bone Miner Res (2013) 28(11):2392–9. 10.1002/jbmr.1976 23661628

[149] Prudowsky ZD Yustein JT. Recent Insights into Therapy Resistance in Osteosarcoma. Cancers (Basel) (2020) 13(1):83. 10.3390/cancers13010083 PMC7795058 33396725

[150] Liu Y Liao S Bennett S Tang H Song D Wood D. STAT3 and its targeting inhibitors in osteosarcoma. Cell Prolif (2020) 54(2):e12974. 10.1111/cpr.12974 33382511 PMC7848963

[151] Oliveira ID Petrilli AS Tavela MH Zago MA de Toledo SR. TNF-alpha, TNF-beta, IL-6, IL-10, PECAM-1 and the MPO inflammatory gene polymorphisms in osteosarcoma. J Pediatr Hematol Oncol (2007) 29(5):293–7. 10.1097/MPH.0b013e3180587e69 17483704

[152] Tuohy JL Somarelli JA Borst LB Eward WC Lascelles BDX Fogle JE. Immune dysregulation and osteosarcoma: Staphylococcus aureus downregulates TGF-beta and heightens the inflammatory signature in human and canine macrophages suppressed by osteosarcoma. Vet Comp Oncol (2020) 18(1):64–75. 10.1111/vco.12529 31420936 PMC7384208

[153] Xiao Q Zhang X Wu Y Yang Y. Inhibition of macrophage polarization prohibits growth of human osteosarcoma. Tumour Biol (2014) 35(8):7611–6. 10.1007/s13277-014-2005-y 24798973

[154] Zhou Q Xian M Xiang S Xiang D Shao X Wang J. All-Trans Retinoic Acid Prevents Osteosarcoma Metastasis by Inhibiting M2 Polarization of Tumor-Associated Macrophages. Cancer Immunol Res (2017) 5(7):547–59. 10.1158/2326-6066.CIR-16-0259 28515123

[155] Han Q Shi H Liu F. CD163(+) M2-type tumor-associated macrophage support the suppression of tumor-infiltrating T cells in osteosarcoma. Int Immunopharmacol (2016) 34:101–6. 10.1016/j.intimp.2016.01.023 26938675

[156] Spencer GJ Utting JC Etheridge SL Arnett TR Genever PG. Wnt signalling in osteoblasts regulates expression of the receptor activator of NFkappaB ligand and inhibits osteoclastogenesis in vitro. J Cell Sci (2006) 119(Pt 7):1283–96. 10.1242/jcs.02883 16522681

[157] Weivoda MM Ruan M Hachfeld CM Pederson L Howe A Davey RA. Wnt Signaling Inhibits Osteoclast Differentiation by Activating Canonical and Noncanonical cAMP/PKA Pathways. J Bone Miner Res (2019) 34(8):1546–8. 10.1002/jbmr.3740 31415114

[158] Glass DA2 Bialek P Ahn JD Starbuck M Patel MS Clevers H. Canonical Wnt signaling in differentiated osteoblasts controls osteoclast differentiation. Dev Cell (2005) 8(5):751–64. 10.1016/j.devcel.2005.02.017 15866165

[159] Yao Y Cai X Yu H Xu Q Li X Yang Y. PSTPIP2 attenuates joint damage and suppresses inflammation in adjuvant-induced arthritis. Eur J Pharmacol (2019) 859:172558. 10.1016/j.ejphar.2019.172558 31325437

[160] Yao Y Yu H Liu Y Xu Q Li X Meng X. PSTPIP2 Inhibits the Inflammatory Response and Proliferation of Fibroblast-Like Synoviocytes in vitro. Front Pharmacol (2018) 9:1432. 10.3389/fphar.2018.01432 30564127 PMC6289071

[161] Hasegawa T Kikuta J Sudo T Matsuura Y Matsui T Simmons S. Identification of a novel arthritis-associated osteoclast precursor macrophage regulated by FoxM1. Nat Immunol (2019) 20(12):1631–43. 10.1038/s41590-019-0526-7 31740799

[162] Umar S Palasiewicz K Van Raemdonck K Volin MV Romay B Ahmad I. CCL25 and CCR9 is a unique pathway that potentiates pannus formation by remodeling RA macrophages into mature osteoclasts. Eur J Immunol (2020). 10.1002/eji.202048681 PMC10041658 33347617

[163] de la Rica L Garcia-Gomez A Comet NR Rodriguez-Ubreva J Ciudad L Vento-Tormo R. NF-kappaB-direct activation of microRNAs with repressive effects on monocyte-specific genes is critical for osteoclast differentiation. Genome Biol (2015) 16:2. 10.1186/s13059-014-0561-5 25601191 PMC4290566

[164] Komano Y Nanki T Hayashida K Taniguchi K Miyasaka N. Identification of a human peripheral blood monocyte subset that differentiates into osteoclasts. Arthritis Res Ther (2006) 8(5):R152. 10.1186/ar2046 16987426 PMC1779441

[165] Chen W Zhu G Jules J Nguyen D Li YP. Monocyte-Specific Knockout of C/ebpalpha Results in Osteopetrosis Phenotype, Blocks Bone Loss in Ovariectomized Mice, and Reveals an Important Function of C/ebpalpha in Osteoclast Differentiation and Function. J Bone Miner Res (2018) 33(4):691–703. 10.1002/jbmr.3342 29149533 PMC6240465

[166] Dapunt U Maurer S Giese T Gaida MM Hansch GM. The macrophage inflammatory proteins MIP1alpha (CCL3) and MIP2alpha (CXCL2) in implant-associated osteomyelitis: linking inflammation to bone degradation. Mediators Inflamm (2014) 2014:728619. 10.1155/2014/728619 24795505 PMC3984830

[167] Jiang P Gao W Ma T Wang R Piao Y Dong X. CD137 promotes bone metastasis of breast cancer by enhancing the migration and osteoclast differentiation of monocytes/macrophages. Theranostics (2019) 9(10):2950–66. 10.7150/thno.29617 PMC6568184 31244935

[168] Ammari M Presumey J Ponsolles C Roussignol G Roubert C Escriou V. Delivery of miR-146a to Ly6C(high) Monocytes Inhibits Pathogenic Bone Erosion in Inflammatory Arthritis. Theranostics (2018) 8(21):5972–85. 10.7150/thno.29313 PMC6299444 30613275

[169] Mann M Barad O Agami R Geiger B Hornstein E. miRNA-based mechanism for the commitment of multipotent progenitors to a single cellular fate. Proc Natl Acad Sci U S A (2010) 107(36):15804–9. 10.1073/pnas.0915022107 PMC2936650 20720163

